# CD24-targeted cystine and glucose oxidase cascade catalytic nanosystem triggers disulfidptosis in neuroblastoma

**DOI:** 10.1016/j.mtbio.2025.102496

**Published:** 2025-11-01

**Authors:** Tao Mi, Junhong Liu, Junyi Luo, Xiangpan Kong, XiaoJun Tan, Liming Jin, Peng Guo, Dawei He

**Affiliations:** a Department of Urology, Children’s Hospital of Chongqing Medical University, National Clinical Research Center for Child Health and Disorders, Ministry of Education Key Laboratory of Child Development and Disorders, Children Urogenital Development and Tissue Engineering of Chongqing Education Commission of China, The Laboratory of Targeted Delivery of Traditional Chinese Medicine, China; b The Second Affiliated Hospital of Chongqing Medical University, Key Laboratory of Integrated Therapy of Traditional Chinese Medicine for Tumors, Chongqing Municipal Administration of Traditional Chinese Medicine, China; c Beijing Anzhen Nanchong Hospital, Capital Medical University & Nanchong Central Hospital, The Second Clinical Medical College of North Sichuan Medical College, China; d Institute of Basic Medicine and Cancer (IBMC), Chinese Academy of Sciences, Hangzhou, Zhejiang, 310022, China

**Keywords:** Disulfidptosis, Glucose oxidase, Cystine, CD24, Neuroblastoma

## Abstract

Neuroblastoma remains a challenging pediatric malignancy with limited therapeutic options, often complicated by chemoresistance and severe systemic toxicity. In this study, we developed a CD24-targeted nanodrug delivery platform that co-delivers cystine and glucose oxidase (GOx) to induce disulfidptosis in neuroblastoma cells. We engineered exosome-mimetic vesicles (EM-CD24) by transfecting HEK-293T cells with a plasmid encoding an anti-CD24 nanobody fused to a glycosylphosphatidylinositol (GPI) anchor signal derived from decay-accelerating factor (DAF), followed by sequential extrusion to obtain EMs with native exosome-like properties and scalable production potential. These vesicles display surface anti-CD24 nanobodies, enabling tumor-specific targeting. Our findings revealed that while cystine promotes cell growth under normal conditions, it induces disulfidptosis under glucose-deprived conditions. Leveraging this metabolic duality, we developed a redox-responsive nanoplatform, Cys-hMnO_2_@GOx@EM-CD24, by co-loading cystine and GOx into hollow MnO_2_ nanoparticles and encapsulating them within EM-CD24 vesicles. CD24-mediated targeting significantly enhanced drug accumulation at the tumor site, reduced NADPH levels, and triggered cystine-induced disulfidptosis. This strategy markedly suppressed both primary and metastatic tumor growth with minimal systemic toxicity. Our findings highlight the efficacy of CD24-guided delivery and demonstrate the translational potential of exploiting tumor metabolic vulnerabilities through environment-responsive nanotherapeutics.

## Highlights

• Identified CD24 as a therapeutic target in neuroblastoma and built CD24-targeted exosome mimetics.
• Revealed cystine’s dual role: promotes growth with glucose, triggers disulfidptosis when glucose is limited.
• Demonstrated the potential of combined GOx and cystine treatment to induce disulfidptosis in tumor cells.
• Developed Cys-hMnO_2_@GOx@EM-CD24 nanoplatform for GOx/cystine co-delivery to induce disulfidptosis.

## Graphical abstract

**Figure:**
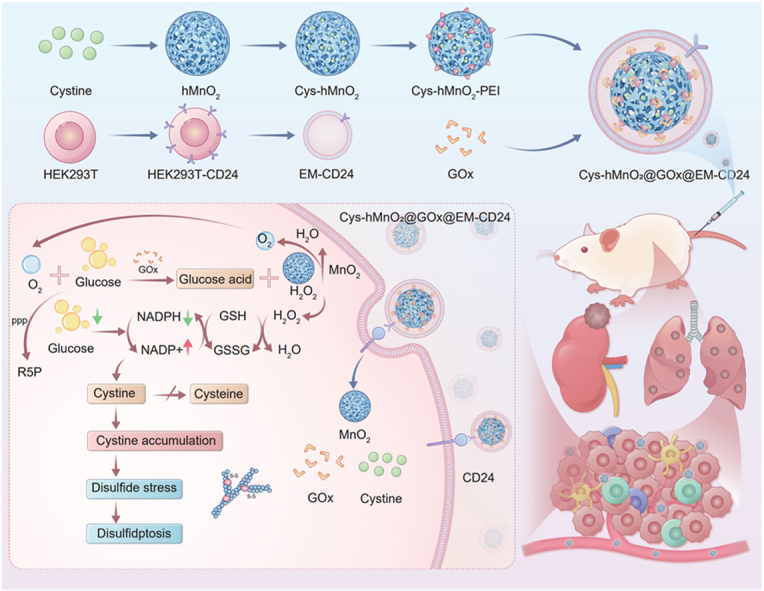

Neuroblastoma is the most common extracranial solid tumor in children [1]. Current treatment strategies primarily rely on surgery combined with chemotherapy and radiotherapy [2]. However, chemotherapeutic regimens are frequently limited by myelosuppression [3,4], hepatic and renal toxicity [5,6], and a lack of responsiveness in high-risk patients, whose 5-year survival rate remains below 50 % [1,4]. These limitations highlight the urgent need for more effective and safer therapeutic strategies. Targeted drug delivery systems offer a promising approach to enhance therapeutic efficacy while minimizing systemic toxicity [7,8].

Exosomes (Exos), small extracellular vesicles ranging from 30 to 150 nm in size, are naturally secreted nanocarriers involved in intercellular communication, and possess excellent biocompatibility and macromolecule delivery capacity [[9], [10], [11]]. Owing to their intrinsic homing ability, exosomes can accumulate and preferentially target tissues of origin. Moreover, exosomes express high levels of the “don't eat me” signal CD47, enabling them to evade phagocytosis and extend circulation time in vivo [12,13]. However, native exosomes still lack active targeting specificity. Receptor-ligand interaction-based targeting, particularly antibody–antigen recognition, has emerged as a powerful strategy to enhance delivery precision [14,15]. Previous studies have engineered exosomes by fusing targeting ligands to exosomal membrane proteins such as Lamp2b [16]. Notably, Sander et al. show that nanobodies can be anchored on the surface of EVs via GPI, which alters their cell targeting behaviour [17]. In our previous work, we identified CD24 as a highly glycosylated and variably expressed protein overexpressed in various tumors and tumor-initiating cells [18,19]. CD24 is highly expressed in neuroblastoma and correlates with poor clinical prognosis [20]. Then, we engineered HEK-293T cells to stably express anti-CD24 nanobodies anchored via the DAF-derived GPI signal peptide. Exosome-mimetic vesicles (EMs) displaying anti-CD24 nanobodies were then fabricated via sequential extrusion and validated in vitro and in vivo for their tumor-targeting capabilities.

However, despite continuous advancements in nanocarrier technologies, the therapeutic efficacy of chemotherapy against tumors remains limited [21]. Nanoparticle-based formulations of chemotherapeutic agents, such as doxorubicin, have seen limited clinical application in neuroblastoma. This highlights the intrinsic limitations of conventional cytotoxic drugs, even when delivered via targeted systems. These findings underscore the urgent need to move beyond traditional chemotherapy. Within this context, our focus shifted toward an emerging form of regulated cell death—disulfidptosis, a recently discovered form of cell death triggered by cystine metabolism dysregulation [[22], [23], [24], [25]]. Tumor cells frequently overexpress the cystine/glutamate antiporter SLC7A11 to facilitate excessive cystine uptake for glutathione (GSH) biosynthesis—an essential antioxidant defense and contributor to chemoresistance [[26], [27], [28]]. This process consumes large amounts of NADPH to reduce cystine to cysteine [22]. As NADPH is mainly produced via the pentose phosphate pathway (PPP), which is glucose-dependent, glucose deprivation leads to NADPH depletion, impaired cystine reduction, and abnormal disulfide bond formation between cytoskeletal proteins, culminating in F-actin collapse and cell death [29,30]. Interestingly, our studies revealed a metabolic duality of cystine: it supports cell growth under normoglycemic conditions but promotes disulfidptosis when glucose is scarce. This context-dependent toxicity underscores cystine's therapeutic potential and safety profile when combined with targeted delivery.

To this end, we developed a multifunctional, cascade-activated nanoplatform (Cys–hMnO_2_@GOx@EM-CD24) based on two design principles: (1) Core architecture: hollow manganese dioxide (hMnO_2_) nanoparticles co-loaded with GOx and cystine. GOx depletes glucose and generates H_2_O_2_ [[31], [32], [33], [34]], while MnO_2_ catalyzes H_2_O_2_ decomposition into O_2_, alleviating hypoxia and sustaining GOx activity [34,35]. Exogenous cystine bypasses the limits of SLC7A11 transport and directly induces disulfidptosis. (2) Targeted delivery: We engineered EMs expressing anti-CD24 single-chain variable fragments (scFvs), with the nanobody anchored to the membrane surface via a glycosylphosphatidylinositol (GPI) anchor. This membrane localization ensured stable surface display and effectively altered the targeting behavior of the EMs (see Scheme 1). This strategy achieves therapeutically relevant intratumoral cystine concentrations with minimal systemic exposure and off-target toxicity.

**Scheme 1:**
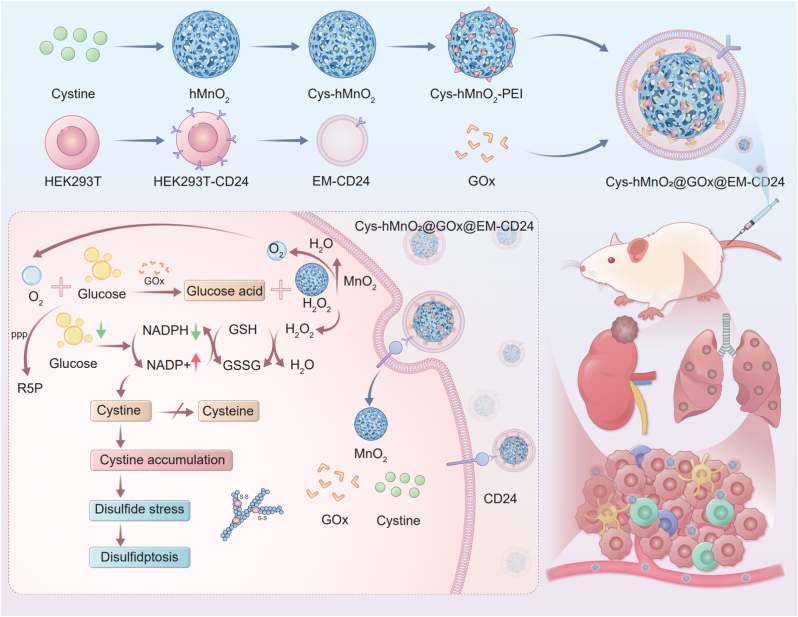
**Schematic illustration of Cys-hMnO_2_@GOx@EM-CD24 mediated tumor cell disulfidptosis.** A hollow manganese dioxide (hMnO_2_) framework served as a metallic scaffold for cystine loading, followed by polyethylenimine (PEI) surface functionalization to yield **Cys-hMnO_2_-PEI**. Lentiviral transfection was employed to engineer HEK293T cells expressing anti-CD24 single-domain antibodies, which were subsequently subjected to extrusion-based nanovesiculation to generate CD24-targeted exosome mimetics (EM-CD24). The final nanocomposite (Cys-hMnO_2_@GOx@EM-CD24) was constructed by conjugating glucose oxidase (GOx) onto Cys-hMnO_2_-PEI followed by EM-CD24 coating. Upon intravenous administration, the nanocomposite selectively accumulates in tumor tissues. In the tumor microenvironment, GOx depletes glucose, leading to NADPH exhaustion. This depletion impairs the reduction of cystine to cysteine, leading to cystine accumulation, redox collapse, and ultimately disulfidptosis through lethal disulfide stress.

In summary, this nanoplatform integrates metabolic interference, targeted delivery, and intelligent release to achieve a robust and selective induction of disulfidptosis. This strategy not only enhances therapeutic efficacy but also improves safety, representing a novel paradigm for the treatment of solid tumors.

## 1 Result

### 1.1 Engineering and functional validation of CD24-targeted nanovesicles

**Fig. 1:**
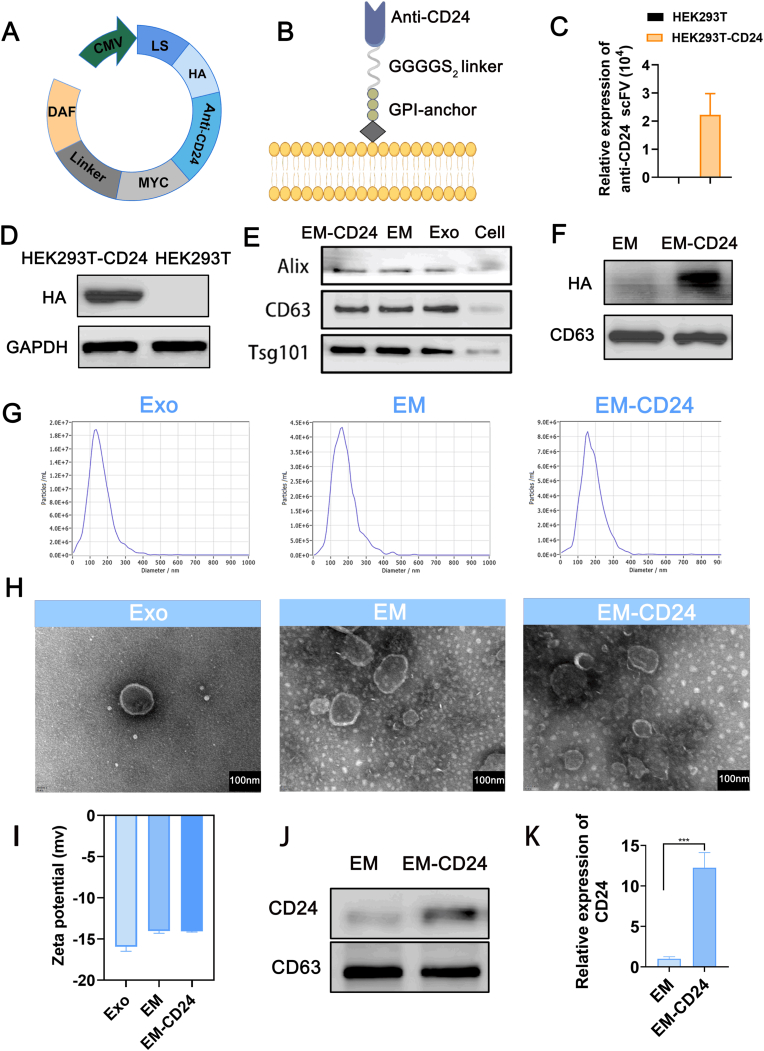
**The production of CD24-targeted engineered exosome mimics (EM-CD24).** (A) The nanobody-DAF sequence was cloned into a pLNCX vector under a CMV promoter, encoding an Igκleader (LS), HA tag, nanobody, Myc tag, GGGGS_2_linker, and a C-terminal GPI-anchor signal peptide (DAF). (B) Upon expression, GPI-anchored nanobodies were expected to localize to lipid rafts in the cell membrane. (C) RT-PCR confirming scFv transcript expression (mean ± SD, n = 3). (D) The expression of tag proteins (HA) was detected by WB. (E–F) EM-CD24 characterization: Classical exosomal markers (CD63/TSG101/Alix) (E) and HA (F) was detected by WB. (G) Nanoparticle tracking analysis revealing monodisperse size distribution. (H) Transmission electron microscopy (TEM) revealed the well-preserved ultrastructure of EM-CD24. (I) Zeta potential analysis (mean ± SD, n = 3). (J–K) The SK-N-DZ cell lysate was incubated with EM-CD24, and the ability of EM-CD24 to bind CD24 protein was detected by WB (J) Corresponding statistical bar chart data (K) (mean ± SD, n = 3). Statistical comparisons by one-way ANOVA with Tukey's post hoc; ∗∗∗ represents P < 0.001.

To validate CD24 as a feasible therapeutic target in neuroblastoma, we examined its expression in clinical specimens and neuroblastoma cell lines. The results demonstrated that CD24 was highly expressed and predominantly localized on the cell membrane (Fig. S1A–F). Functional assays further demonstrated that CD24 knockdown suppresses neuroblastoma cell proliferation, invasion, and migration (Fig. S2A–G). We then generated EM-CD24 following the workflow illustrated in Fig. S2H. HEK293T-CD24 cells were generated by lentiviral transduction of 293T cells with a construct encoding the anti-CD24 antibody display sequence. This sequence was cloned into a vector designed to co-express Myc and HA tags fused to the DAF polypeptide, enabling membrane anchoring and subsequent detection (Fig. 1A–B). RT-PCR using specific primers designed for the anti-CD24 scFv sequence confirmed successful expression in the engineered HEK-293T cells (HEK293T-CD24) (Fig. 1C). Western blot analysis further validated the expression of the HA-tagged fusion protein, confirming efficient transduction (Fig. 1D). Engineered vesicles (EM-CD24) were then produced by extruding HEK293T-CD24 cells through a lipid extruder [36]. Western blot analysis confirmed that EM-CD24 carried classical exosomal markers such as CD63, TSG101, and Alix, consistent with the proteomic profile of native exosomes (Fig. 1E). Importantly, the presence of HA-tagged protein in EM-CD24 validated the successful inheritance of anti-CD24 scFv from donor cells (Fig. 1F). The average diameter of EM-CD24 vesicles was approximately 160 nm, slightly larger than that of native exosomes (134 nm) (Fig. 1G). Scanning electron microscopy further confirmed the structural integrity of the vesicles, revealing typical spherical, bilayered membrane morphology in EM-CD24 preparations (Fig. 1H). Zeta potential measurements indicated that both EM-CD24 and native exosomes bore a negative surface charge, ranging from −10 mV to −15 mV (Fig. 1I). Functional validation demonstrated superior targeting specificity: when incubated with SK-N-DZ cell lysates, EM-CD24 exhibited over 10-fold greater CD24-capturing capacity compared to non-targeted vesicles, confirming precise antibody-mediated targeting (Fig. 1J–K).

### 1.2 Cys-hMnO2@GOx@EM-CD24 construction and characterization

**Fig. 2:**
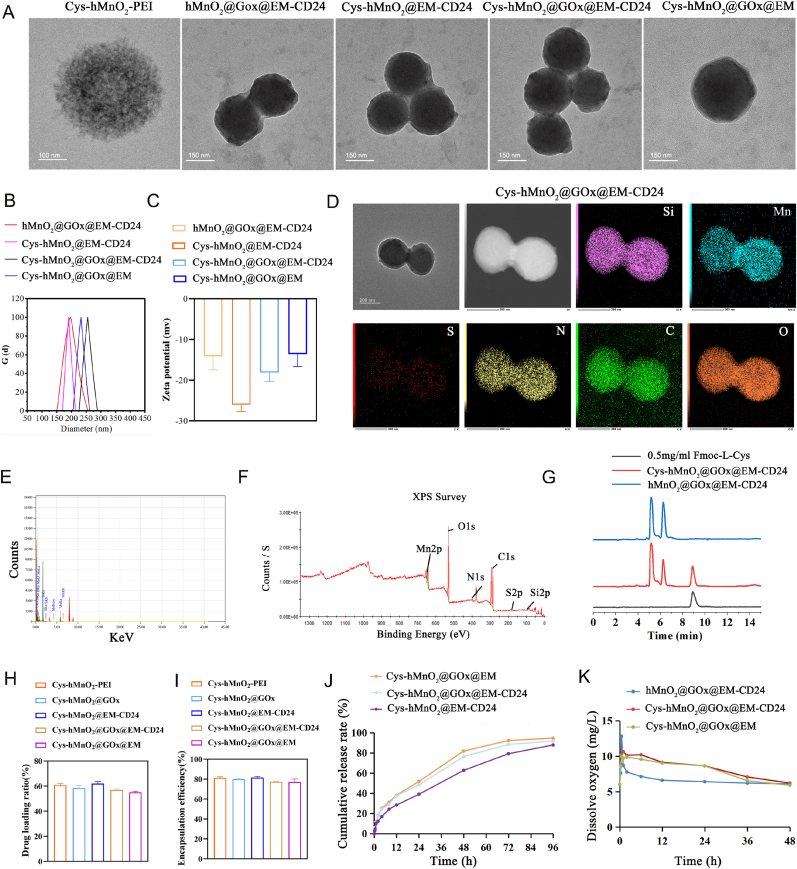
**Synthesis and characterization of Cys-MnO_2_@GOx@EM-CD24.** (A) Transmission electron microscopy (TEM) images demonstrated uniform spherical morphology and nanoscale structure. (B) Dynamic light scattering (DLS) analysis revealed the hydrodynamic diameter of the nanosystem (mean ± SD, n = 3). (C) Zeta potential measurements confirmed surface charge properties (mean ± SD, n = 3). (D–E) Energy-dispersive X-ray spectroscopy (EDS) elemental mapping and quantification verified the spatial distribution and relative abundance of key elements within the nanocomposite. (F) X-ray photoelectron spectroscopy (XPS) provided atomic-level insights into surface chemical states and functional bond configurations, supporting successful loading (Cys-hMnO_2_-PEI). (G–I) High-performance liquid chromatography (HPLC) was used to quantify cystine loading content and encapsulation efficiency (mean ± SD, n = 3). (J) In vitro cystine release kinetics (mean, n = 3). (K) Oxygen generation was evaluated to confirm MnO_2_-mediated catalytic decomposition of H_2_O_2_, validating the nanoplatform's capacity to modulate hypoxia (mean, n = 3).

Our study revealed a metabolic duality of cystine: it maintains viability and promotes proliferation of neuroblastoma cells under normoglycemic conditions (<400 μM) but triggers disulfidptosis when glucose is scarce (Figs. S3–S4). Building on this finding, we demonstrated that co-administration of glucose oxidase (GOx) and cystine effectively induces disulfidptosis in neuroblastoma cells (Fig. S5). However, the poor solubility of cystine and the systemic side effects of GOx hinder their direct in vivo application [[31], [32], [33],^37^]. To overcome these challenges, we developed a nanoplatform, Cys-hMnO_2_@GOx@EM-CD24, for efficient and safe in vivo delivery. Cys-hMnO2@GOx@EM-CD24 was prepared as described in the Methods section. Briefly, Cys-MnO_2_-PEI nanoparticles were prepared, after which glucose oxidase (GOx) was loaded, and the final construct was encapsulated with EM-CD24. High-resolution TEM revealed that Cys-MnO2-PEI nanoparticles exhibit a hollow architecture with a crystalline core and porous shell, which transitioned to solid core-shell morphologies after sequential GOx and EM functionalization, confirming successful surface modification (Fig. 2A). Dynamic light scattering analysis revealed that the Cys-MnO_2_@GOx@EM-CD24 exhibited a diameter ranging from 150 to 300 nm, which was slightly larger than that of drug-free EM-CD24 (Fig. 2B). Zeta potential measurements indicated that the particles carried a negative surface charge (−18.06 ± 2.18 mV) with the addition of EM-CD24 (Fig. 2C). Energy-dispersive X-ray spectroscopy (EDS) elemental mapping confirmed homogeneous distribution of Mn (14.27 wt%), O (19.12 wt%), C (31.11 wt%), N (1.5 wt%), and S (0.06 wt%) (Fig. 2DE). X-ray photoelectron spectroscopy (XPS) analysis of Cys-hMnO_2_-PEI nanoparticles revealed a surface composition comprising Mn, O, C, N, Si, and S. High-resolution Mn 2p spectra, with peaks at 642.5 eV and 654.1 eV, confirmed Mn^4+^ as the dominant oxidation state, while a 0.6 eV shift in Mn 2p indicated Mn–S coordination from cystine. The S2p spectrum showed 73.5 % of thiol groups bound to the surface, with the remainder forming disulfide bonds. Deconvoluted O1s spectra identified lattice oxygen, hydroxyl groups (34.8 %), and Mn–O–C bonds, with hydroxyls linked to catalytic activity via oxygen vacancies. N1s peaks verified the PEI coating through primary, secondary, and protonated amine signals. (Fig. 2F). To further evaluate the functional performance of the nanocarrier, we next quantified its drug loading and release behavior. High-Performance Liquid Chromatography (HPLC) analysis revealed superior cystine loading in Cys-hMnO2@EM-CD24, with 82.73 % encapsulation efficiency and 63.73 % drug loading, outperforming Cys-hMnO_2_@GOx@EM-CD24 (77.85 %, 56.58 %) and Cys-hMnO2@GOx@EM (76.19 %, 54.63 %) (Fig. 2G–I). Cystine release kinetics exhibited an initial burst (0–10min) with similar rates, followed by accelerated release profile (10min–30min) where GOx-modified nanoparticles (Cys-hMnO_2_@GOx@EM, Cys-hMnO_2_@GOx@EM-CD24) achieved 37–46 % higher release than Cys-hMnO_2_@EM-CD24. Sustained release (0.5–24 h) reached 51.78 % and 48.28 % for GOx-modified systems, surpassing the unmodified system (39.07 %) by 23.6–32.5 %. GOx likely enhances release through enzymatic generation of hydrogen peroxide or local pH modulation, destabilizing the nanoparticle matrix and facilitating cystine desorption (Fig. 2J). Hydrogen peroxide assays demonstrated the nanomaterials’ ability to generate H_2_O_2_, confirming adequate GOx loading (Fig. S6A). Dissolved oxygen assays revealed that hMnO_2_@GOx@EM-CD24 achieved rapid initial O_2_ release (12.80 mg/L at 0.5 h) but suffered rapid decay (7.13 mg/L at 6 h). In contrast, Cys-hMnO_2_@GOx@EM-CD24 maintained stable O_2_ levels (≥9.5 mg/L over 6 h, decay rate <4.7 %) through confined catalytic microenvironments and dynamic cystine-mediated regulation of active site exposure, effectively balancing H_2_O_2_ decomposition and O_2_ generation (Fig. 2K). Stability testing showed that the nanomaterials maintained consistent hydrodynamic size and zeta potential (Fig. S6B–C). Cellular uptake experiments demonstrated the targeted drug delivery capability of the nanosystem. Cys-hMnO_2_@GOx@EM-CD24 showed significantly higher cellular uptake compared to non-targeted vesicles, attributable to antibody–antigen interaction-mediated endocytosis (Fig. S6D).

### 1.3 Cys-hMnO_2_@GOx@EM-CD24 induces disulfidptosis in neuroblastoma cells in vitro

**Fig. 3:**
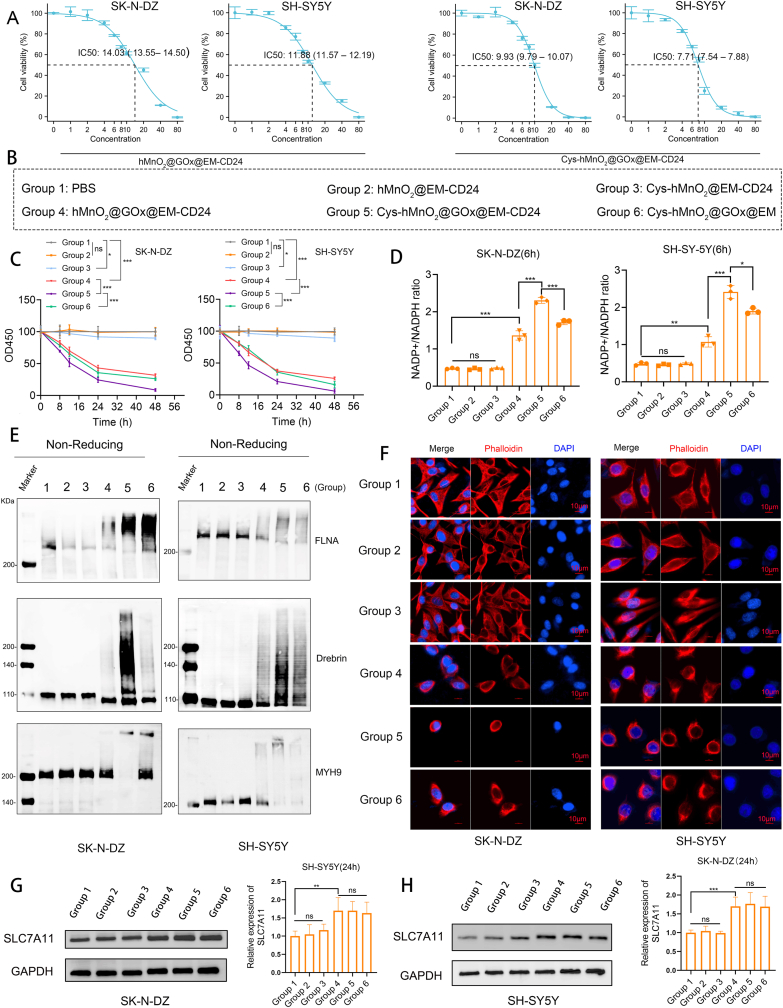
**Cys-hMnO_2_@GOx@EM-CD24 induces disulfidptosis in neuroblastoma cells in vitro.** (A). The IC50 of the nanomaterials was determined using the CCK-8 assay (mean ± SD, n = 5). (B) Six groups were established to evaluate the efficacy of the combination therapy and the targeted modification strategy. (C) Cell viability assay indicating a significant reduction in neuroblastoma cell proliferation following treatment with the nanoplatform (mean ± SD, n = 5). (D) Intracellular NADPH levels were markedly depleted upon treatment (mean ± SD, n = 3). (E) Non-reducing Western blot analysis revealed disulfide-linked aggregation of cytoskeletal proteins FLNA, Drebrin, and MYH9. (F) Immunofluorescence staining of F-actin demonstrated severe cytoskeletal disorganization. (G–H) Western blot analysis showed upregulation of SLC7A11 (mean ± SD, n = 3). 4-parameter logistic fit for IC50 (A). Two-way ANOVA with Tukey's post hoc (C). One-way ANOVA with Tukey's post hoc (D,G,H); ns represents P > 0.05, ∗ represents P < 0.05, ∗∗ represents P < 0.01, ∗∗∗ represents P < 0.001.

To rigorously assess the therapeutic potential of the combined nano-delivery platform, we first performed CCK-8 assays to determine the half-maximal inhibitory concentration (IC_50_) of the treatment group (Cys-hMnO_2_@GOx@EM-CD24). For comparative purposes, the IC_50_ of the GOx-only formulation (hMnO_2_@GOx@EM-CD24) was also measured. As shown in Fig. 3A, Cys-hMnO_2_@GOx@EM-CD24 exhibited a significantly lower IC_50_ value (SK-N-DZ: 9.93 μg/mL; SH-SY5Y: 7.71 μg/mL) compared to hMnO_2_@GOx@EM-CD24 (SK-N-DZ: 14.03 μg/mL; SH-SY5Y: 11.88 μg/mL), indicating its markedly enhanced cytotoxic efficacy. To further compare the therapeutic efficacy across groups, hMnO_2_@GOx@EM-CD24 was used as the positive control. Its 2 IC_50_ values were adopted as fixed total dosages for subsequent in vitro experiments. Six experimental groups were established to systematically evaluate the role of each constituent: PBS (Group 1), hMnO_2_@EM-CD24 (Group 2), Cys-hMnO_2_@EM-CD24 (Group 3), hMnO_2_@GOx@EM-CD24 (Group 4), Cys-hMnO_2_@GOx@EM-CD24 (Group 5), and Cys-hMnO_2_@GOx@EM (Group 6) (Fig. 3B). hMnO_2_@EM-CD24 alone exhibited no cytotoxic effects, confirming its biosafety as a nanocarrier material. Interestingly, treatment with Cys-hMnO_2_@EM-CD24 slightly suppressed cell proliferation; however, exogenous cystine alone may be insufficient to induce disulfidptosis. Therefore, caution is warranted, and co-administration with a NADPH-depleting strategy may be necessary to achieve the desired effect. hMnO_2_@GOx@EM-CD24 demonstrated a moderate cytotoxic effect. Notably, Cys-MnO_2_@GOx@EM-CD24 showed the most pronounced cytotoxicity at equivalent doses (Fig. 3C). We also observed that the tumor-suppressive efficacy of nanomaterials encapsulated in unmodified EM was inferior to that of those delivered via anti-CD24-modified EM, underscoring the necessity of targeted surface modification to enhance therapeutic performance (Fig. 3C).

The extent of NADPH depletion was proportional to the observed level of cell death, providing preliminary evidence supporting the occurrence of disulfidptosis (Fig. 3D). Moreover, we observed coordinated redox changes: ROS increased as NADPH decreased, and the GSH/GSSG ratio declined accordingly (Fig. S7A–D). Non-reducing Western blot analyses confirmed the presence of disulfidptosis-specific biomarkers, including high-molecular-weight aggregates of MYH9, Drebrin, and FLNA (Fig. 3E, Figure S7E), thereby validating the mode of cell death. Additionally, phalloidin staining revealed extensive cytoskeletal disintegration (Fig. 3F). Interestingly, GOx-mediated glucose deprivation induced compensatory upregulation of SLC7A11 expression (Fig. 3G,H), a well-established resistance mechanism in the context of apoptosis and ferroptosis. However, within our therapeutic framework, this adaptive response paradoxically enhanced treatment efficacy: the upregulation of SLC7A11 promoted cystine import under conditions of NADPH depletion, further intensifying disulfide accumulation and ultimately driving cytoskeletal collapse and cell death.

### 1.4 In vivo targeting efficacy of Cys-hMnO_2_@GOx@EM-CD24 nanovesicles

**Fig. 4:**
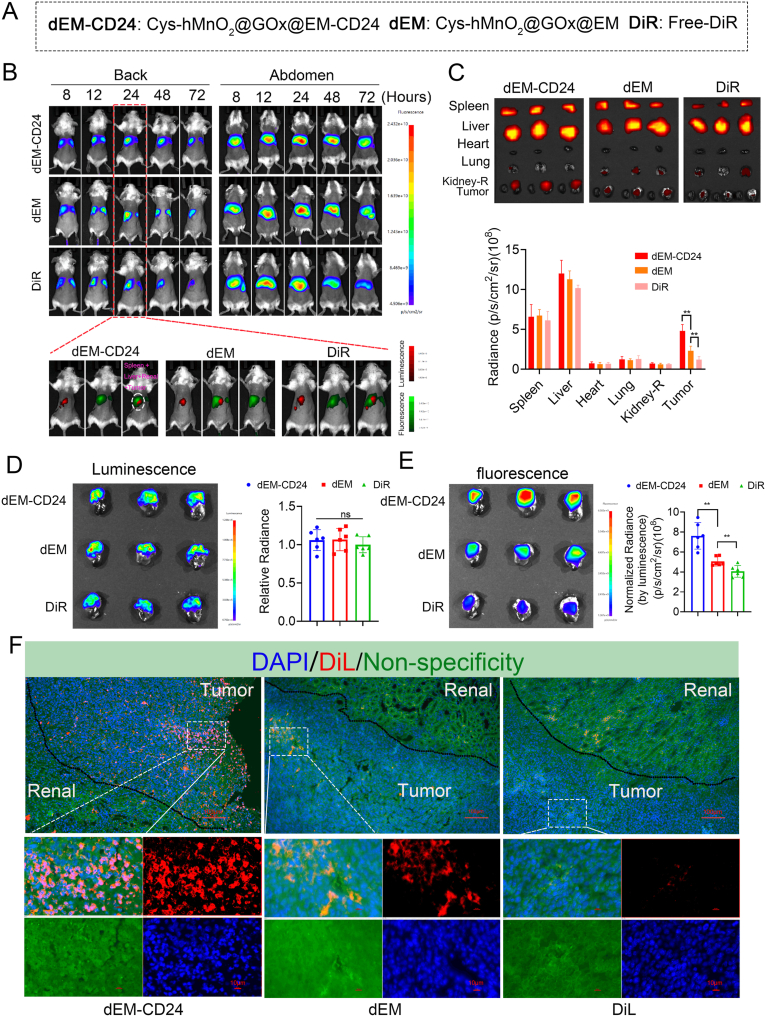
**Cys-hMnO_2_@GOx@EM-CD24 targets orthotopic tumors in mice.** (A) DiR-labeled Cys-hMnO_2_@GOx@EM-CD24 (dEM-CD24), Cys-hMnO_2_@GOx@EM (dEM), and free DiR were used to evaluate targeting specificity. (B) Biodistribution of DiR-labeled dEM-CD24, dEM, and free DiR in mice. (C) Fluorescence distribution and intensity quantification in ex vivo organs (24h) (mean ± SD, n = 6 mice/group). (D) Luciferase (LUC) bioluminescence in orthotopic tumor models (mean ± SD, n = 6 mice/group). (E) Fluorescence distribution and intensity analysis of orthotopic tumor (mean ± SD, n = 6 mice/group). (F) The tissue section displaying DiL and DiL-labeled EM-CD24 distribution (red). Non-specific fluorescence in sections better delineates tumor-kidney boundaries (green). Group differences were tested by one-way ANOVA with Tukey's post hoc test. (For interpretation of the references to color in this figure legend, the reader is referred to the Web version of this article.)

To evaluate the tumor-specific accumulation of the nanovesicles, we orthotopically implanted luciferase-expressing SK-N-DZ cells to establish neuroblastoma xenografts that closely recapitulate the native tumor microenvironment. On day 10 post-implantation, bioluminescence imaging confirmed successful tumor establishment. The targeting capability of Cys-hMnO_2_@GOx@EM-CD24 (dEM-CD24) was evaluated by comparing it with the unmodified group (Cys-hMnO_2_@GOx@EM, dEM) and free DiR (Fig. 4A). Mice were intravenously injected with DiR-labeled dEM-CD24, dEM, or free DiR. Whole-body fluorescence imaging revealed progressive hepatic accumulation of all three formulations between 6 and 24 h post-injection, with the strongest signals localized to the liver and spleen. After 24 h, fluorescence intensity began to decline. Although dEM-CD24 and dEM showed comparable hepatic and splenic fluorescence intensities, dEM-CD24 exhibited notably prolonged in vivo retention compared to free DiR (Fig. 4B).

We next focused on fluorescence signals within the tumor region. However, due to anatomical overlap between the orthotopic tumors and major visceral organs (liver/spleen), precise quantification of tumor-specific uptake in vivo proved challenging. Some researchers have misinterpreted the fluorescence observed in the dorsal hepatic and splenic regions of mice as tumor-specific (or renal) accumulation. However, based on both the fluorescence intensity and anatomical location, this interpretation is inaccurate. Therefore, ex vivo fluorescence imaging of excised organs was performed at 24 h post-injection, then the fluorescence intensity of each organ were calculated respectively. Nanoparticles primarily accumulated in the liver and spleen and tumors (Fig. 4C). After correcting tumor volume based on luciferase (LUC) bioluminescence (Fig. 4D), quantitative comparison revealed that orthotopic tumors in the dEM-CD24-treated group exhibited more than a twofold increase in fluorescence intensity compared to those treated with dEM (Fig. 4E), confirming its tumor-selective delivery capability. Histological analysis further supported cellular-level targeting specificity: dEM-CD24 accumulation (DiL-labeled) was markedly greater in tumor tissues than in adjacent renal tissue (Fig. 4F).

### 1.5 Cys-hMnO_2_@GOx@EM-CD24 suppress tumor growth

**Fig. 5:**
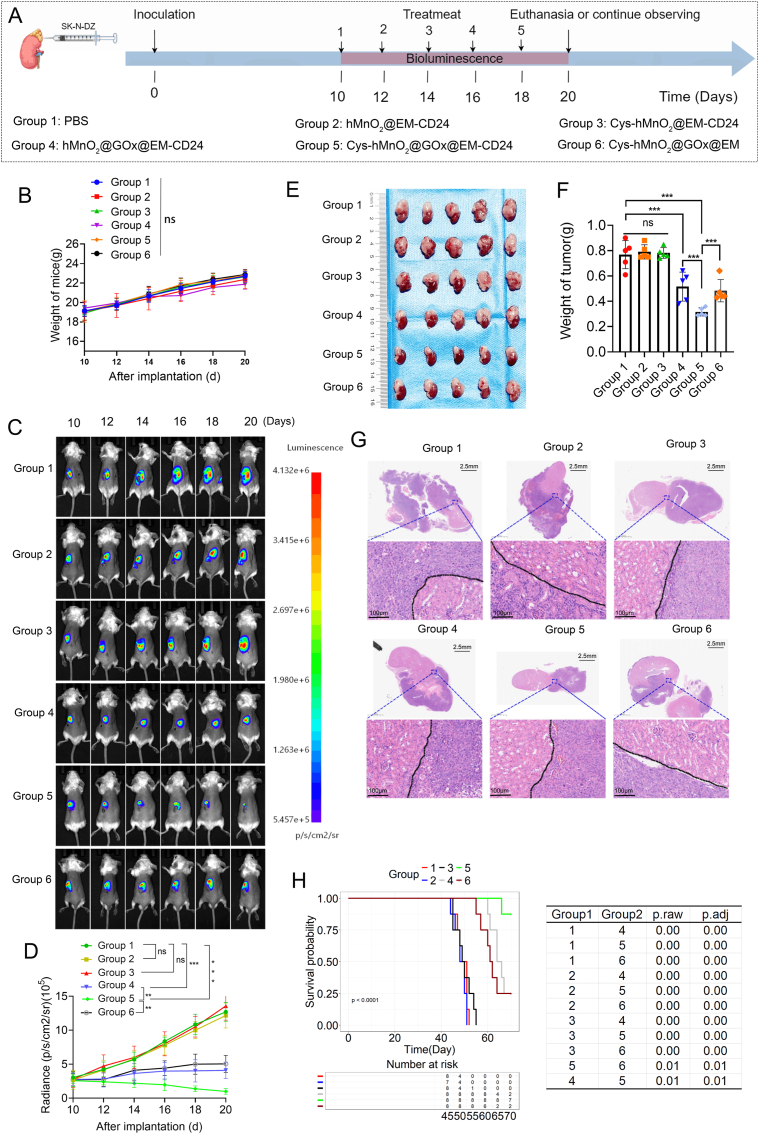
**Therapeutic Evaluation of Cys-hMnO_2_@GOx@EM-CD24 in Orthotopic Neuroblastoma Models.** (A) Schematic workflow of orthotopic tumor model establishment and therapeutic intervention protocol. (B) Longitudinal monitoring of body weight changes during treatment (n = 5 mice/group, mean ± SD). (C) Real-time tumor progression tracking via luciferase (LUC) bioluminescence imaging. (D) Quantitative analysis of tumor-associated bioluminescence intensity dynamics (n = 5 mice/group, mean ± SD). (E) Gross morphological evaluation of excised orthotopic tumors post-treatment. (F) Quantification of orthotopic tumor weight (n = 5 mice/group, mean ± SD). (G) Hematoxylin and eosin (H&E) staining for histopathological assessment of tumor tissues. (H) Kaplan-Meier survival curves across treatment cohorts (n = 8). Repeated-measures ANOVA for (B,D); one-way ANOVA with Tukey for (F); log-rank test for (H). ns represents P > 0.05, ∗∗∗ represents P < 0.001.

To assess the antitumor efficacy of the nanodrug, we established a tumor-bearing mouse model as illustrated in Fig. 5A and administered treatments accordingly. Tumor progression was monitored via in vivo bioluminescence imaging, while body weight was recorded throughout to evaluate systemic toxicity. No significant weight loss was observed in any treatment group, indicating favorable biocompatibility and low systemic toxicity (Fig. 5B). Bioluminescent imaging revealed that tumors in the PBS control and hMnO_2_@EM-CD24-treated groups continued to grow, reflecting the natural course of tumor progression. Cystine alone showed no inhibitory effect on tumor growth and did not promote tumor development. In contrast, glucose oxidase (GOx) monotherapy (hMnO_2_@GOx@EM-CD24, Group 4) exhibited moderate antitumor activity, which was markedly enhanced when co-administered with cystine, confirming their synergistic therapeutic effect. Notably, CD24-targeted vesicles demonstrated superior tumor suppression compared to non-targeted formulations, which can be attributed to enhanced tumor accumulation mediated by CD24 antibody-directed targeting (Fig. 5CD). Ex vivo tumor measurements visually confirmed significant tumor regression in the combination therapy group (Fig. 5E), with tumor weight data providing consistent evidence of efficacy (Fig. 5F). Hematoxylin and eosin (H&E) staining confirmed the successful establishment of the orthotopic tumor model and therapeutic efficacy, with a clearly demarcated boundary observed between the tumor and adjacent renal tissue (Fig. 5G). After completion of treatment, another cohort of tumor-bearing mice was allowed to grow naturally to evaluate long-term survival. Mice treated with Cys-hMnO_2_@GOx@EM-CD24 exhibited the longest survival, whereas all mice in the PBS, hMnO_2_@EM-CD24, and Cys-hMnO_2@_EM-CD24 groups died within 60 days. Postmortem analysis revealed that mortality was primarily due to tumor invasion beyond the renal capsule, leading to extensive peritoneal metastasis and severe deterioration in nutritional status (Fig. 5H). Compared with the clinically used agent doxorubicin, Cys-hMnO_2_@GOx@EM-CD24 likewise demonstrated therapeutic efficacy; with comparable body-weight trajectories, the nanoplatform delivered superior tumor control (Fig. S8A–C).

### 1.6 Cys-hMnO_2_@GOx@EM-CD24 safely induces disulfidptosis in vivo

**Fig. 6:**
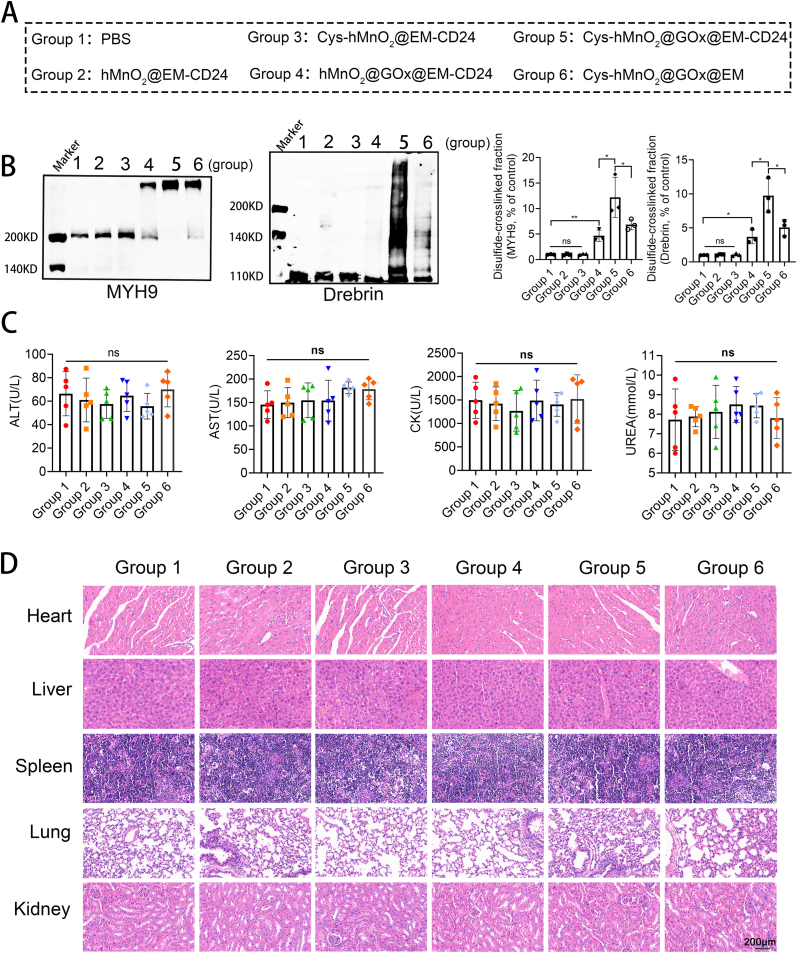
**Mechanistic investigation and biosafety evaluation of Cys-hMnO_2_@GOx@EM-CD24 in an orthotopic neuroblastoma model.** (A–B) Non-reducing Western blot analysis confirmed successful induction of disulfidptosis following treatment (mean ± SD, n = 3). (C) Blood biochemical parameters indicated no signs of systemic toxicity associated with the nanosystem (mean ± SD, n = 5). (D) H&E staining of major organs revealed no significant histological damage across treatment groups. Statistical comparisons by one-way ANOVA with Tukey (B–C). ns represents P > 0.05, ∗ represents P < 0.05, ∗∗ represents P < 0.01, ∗∗∗ represents P < 0.001.

To mechanistically investigate whether disulfidptosis occurred in vivo, we performed non-reducing Western blot analysis. The results demonstrated that Cys-hMnO_2_@GOx@EM-CD24 induced the most prominent disulfidptosis, aligning with the observed tumor growth inhibition, thereby supporting the conclusion that the antitumor effect was mediated by disulfidptosis (Fig. 6A–B). Furthermore, we assessed the expression of proliferation marker PCNA and invasion-associated protein MMP2, both of which were significantly suppressed following treatment with Cys-hMnO2@GOx@EM-CD24 (Fig. S9). To evaluate biosafety, we measured serum biochemical parameters, including Creatine kinase (CK),urea,aspartate aminotransferase (AST), and alanine aminotransferase (ALT), all of which remained within normal physiological ranges (Fig. 6C). Histological analysis of major organs revealed no significant pathological alterations or tissue damage (Fig. 6D). Head-to-head comparison with doxorubicin further underscored the nanomedicine's safety: doxorubicin caused mild hepatic, renal, and cardiac injury and elevated hepatic/renal oxidative-stress indices, whereas Cys-hMnO_2_@GOx@EM-CD24 produced no detectable abnormalities (Fig. S8D–E). To address potential neurotoxicity given CD24 expression in immature neural tissue, we performed focused safety assays: H&E staining of coronal brain sections showed no overt neuropathology (Fig. S10A), and NGF-differentiated PC12 neuron-like cells (CD24^+^, lower than tumor lines) exhibited right-shifted dose–response curves with selectivity indices of ∼3.7 (vs. SK-N-DZ) and ∼4.7 (vs. SH-SY5Y) (Fig. S10B–H). Full data are provided in the Supplementary File 1.

### 1.7 Targeting lung metastases with Cys-hMnO_2_@GOx@EM-CD24

**Fig. 7:**
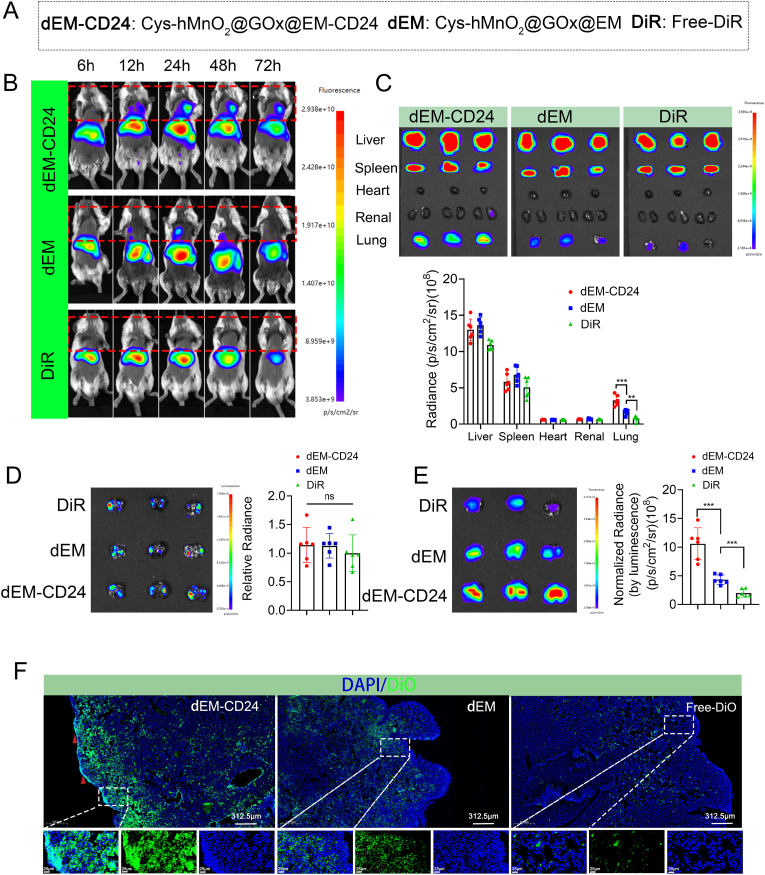
**EM-CD24 targets lung metastases.** (A) DiR-labeled Cys-hMnO_2_@GOx@EM-CD24 (dEM-CD24), Cys-hMnO_2_@GOx@EM (dEM), and free DiR were used to evaluate targeting specificity. (B) Whole-body imaging of DiR-labeled dEM-CD24, dEM,DiR showing systemic distribution. (C) Fluorescence distribution and intensity quantification in ex vivo organs (mean ± SD, n = 6 mice/group). (D) Luciferase bioluminescence in lung metastasis models (mean ± SD, n = 6 mice/group). (E) Fluorescence distribution and intensity analysis of lung metastatic foci. (F) Frozen sections displaying DiO-labeled dEM-CD24 distribution. Statistical comparisons by one-way ANOVA with Tukey post hoc tests for(C, D); ns represents P > 0.05, ∗ represents P < 0.05, ∗∗ represents P < 0.01, ∗∗∗ represents P < 0.001.

Neuroblastoma originates from precursor cells of the sympathetic nervous system, and approximately 50 % of patients present with metastases at the time of diagnosis. Although neuroblastatic lesions may disseminate throughout the body—including the bone marrow, skeleton, lymph nodes, liver, brain, and orbit—lung metastases are associated with the most aggressive progression and poorest prognosis [38,39]. To evaluate the therapeutic potential of Cys-hMnO_2_@GOx@EM-CD24 (dEM-CD24) against pulmonary metastases, we first assessed the lung-targeting ability of the dEM-CD24. A lung metastasis model was established using LUC-labeled SK-N-DZ cells. On day 14, successful model establishment was confirmed, and mice were randomly assigned to receive dEM-CD24, Cys-hMnO_2_@GOx@EM (dEM), or free DiR formulations (Fig. 7A). In vivo fluorescence imaging revealed biodistribution patterns similar to those observed in orthotopic tumor models, with predominant liver and spleen accumulation peaking at 24 h, followed by gradual clearance. Notably, both dEM-CD24 and dEM groups exhibited prolonged systemic retention. Biodistribution analysis revealed that dEM exhibited a certain degree of pulmonary tropism, while dEM-CD24 demonstrated significantly enhanced lung-targeting capability. (Fig. 7B). Ex vivo fluorescence imaging of dissected organs confirmed these findings (Fig. 7C). Upon normalization of metastatic burden via LUC imaging (Fig. 7D), the results showed that dEM-CD24 exhibited fluorescence intensities 2.4-fold higher than the dEM group and 5.3-fold higher than the free DiR group (Fig. 7E).

To further evaluate distribution at the cellular level, another cohort of mice was administered DiO-labeled dEM-CD24, dEM, or free DIO. Consistent with previous findings, the dEM-CD24 group displayed the strongest fluorescence signal, with a distribution pattern closely matching tumor foci (Fig. 7F). These results collectively demonstrate the potent lung metastasis-targeting capability of dEM-CD24, laying a strong foundation for its application in the treatment of pulmonary metastatic neuroblastoma.

### 1.8 Cys-hMnO_2_@GOx@EM-CD24 nanovesicles suppress pulmonary metastasis in neuroblastoma

**Fig. 8:**
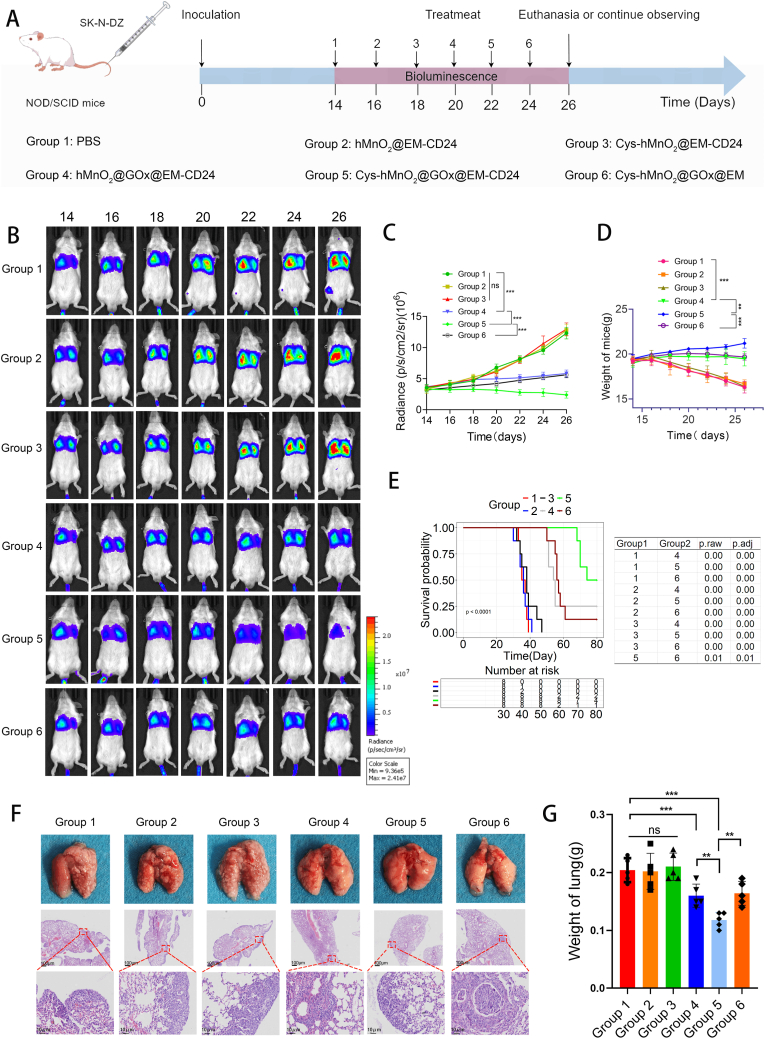
**Evaluation of the therapeutic efficacy of Cys-hMnO_2_@GOx@EM-CD24 in a neuroblastoma lung metastasis model.** (A) Schematic illustration of the establishment of the lung metastasis model and treatment regimen. (B) Bioluminescence imaging to monitor metastatic tumor progression. (C) Quantification of bioluminescence intensity over time (mean ± SD, n = 5 mice/group). (D) Body weight changes of mice during treatment (mean ± SD, n = 5). (E) Kaplan–Meier survival curves showing the survival of mice in different treatment groups (monitored up to 80 days) (n = 8 mice/group). (F) Gross morphology and H&E staining of lung tissues at the end of treatment. (G) Statistical analysis of lung weight in each group on day 26 post-cell inoculation (mean ± SD, n = 5 mice/group). Longitudinal data (C, D) were analyzed by repeated-measures ANOVA; endpoint comparisons (G) by one-way ANOVA with Tukey; survival (E) by log-rank.

Given the potent antitumor efficacy of the constructed nanoparticle system, we further evaluated its ability to suppress tumor metastasis. As shown in Fig. 8A, a pulmonary metastasis model was established by intravenous injection of SK-N-DZ cells into the tail vein of mice. On day 14, successful establishment of lung metastases was confirmed, and mice were randomly divided into six treatment groups. Bioluminescence imaging (BLI) was conducted on days 14, 16, 18, 20, 22, 24, and 26 to monitor tumor progression, and body weight was recorded throughout. BLI results were consistent with those observed in the orthotopic model. Glucose oxidase (GOx) alone exerted moderate antitumor effects, while its combination with cystine further enhanced therapeutic efficacy. Notably, surface modification of the vesicles significantly improved targeting efficiency, thereby strengthening antitumor activity (Fig. 8BC). Unlike orthotopic tumors, pulmonary metastases significantly impacted body weight. However, weight loss was alleviated following treatment, indicating both the efficacy and safety of the formulations (Fig. 8D). These findings confirm the nanoparticles' potent ability to inhibit tumor metastasis. Subsequent survival analysis revealed that mice in the control group succumbed within approximately 40 days, whereas treatment with Cys-hMnO2@GOx@EM-CD24 markedly prolonged survival, further validating its therapeutic potential (Fig. 8E). Ex vivo examination of lung tissues revealed obvious morphological differences between control and treated groups. H&E staining confirmed the presence of prominent metastatic lesions, predominantly located at the lung periphery (Fig. 8F). Lung weight in the control group was significantly elevated due to extensive tumor infiltration, while treated mice showed reduced lung mass, indicative of diminished metastatic burden (Fig. 8F).

In summary, these findings demonstrate the dual therapeutic potential of Cys-hMnO2@GOx@EM-CD24 nanoparticles in suppressing both primary tumor growth and pulmonary metastasis, laying a solid foundation for their future clinical translation in cancer therapy.

## 2 Conclusion

The groundbreaking discovery of disulfidptosis by Gan Boyi's group in 2023 revealed a novel cell death mechanism triggered by synergistic SLC7A11 overexpression and glucose starvation, where excessive cystine influx coupled with NADPH depletion induces catastrophic disulfide stress [22]. This mechanism highlights a unique metabolic vulnerability in tumors, yet its clinical translation is constrained by tumor heterogeneity in SLC7A11 expression and the impracticality of systemic glucose deprivation [23,30]. Our work addresses these challenges through two key innovations: First, we demonstrate that exogenous cystine supplementation bypasses intrinsic SLC7A11 dependency, enabling robust disulfidptosis in SLC7A11 low/intermediate neuroblastomas under glucose-deprived conditions. Second, GOx serves as a dual-function metabolic disruptor: it depletes glucose to suppress NADPH production via the pentose phosphate pathway (PPP) while generating H_2_O_2_ to further oxidize NADPH reserves.

Our study revealed a metabolic duality of cystine: it maintains viability and promotes proliferation of neuroblastoma cells under normoglycemic conditions (<400 μM) but triggers disulfidptosis when glucose is scarce. Here,We also observed that cystine deprivation suppresses cell growth and promotes cell death, with a notable divergence between early and late apoptosis (early apoptosis increased while late apoptosis decreased), consistent with mode switching toward ferroptosis or other non-apoptotic death under low-cystine conditions [40]. Although these data suggest that cystine deprivation also holds antitumor potential, complete cystine withdrawal is difficult to achieve in vivo, and normal cells are highly sensitive to cystine scarcity—sometimes more so than tumor cells. Consequently, choosing high-dose cystine (rather than cystine deprivation) combined with glucose restriction is a more clinically feasible strategy to drive disulfidptosis.

This reasoning reframes the role of glucose oxidase (GOx). Historically, GOx has been used as a metabolic disruptor to deplete glucose and generate ROS, thereby killing tumor cells [33]. In our design, hMnO_2_ provides oxygenation and H_2_O_2_ buffering/control, and adding cystine elevates the mechanism from a single oxidative insult to a dual-pathway lethality—exogenous ROS plus endogenous disulfide stress—which amplifies efficacy. Notably, disulfidptosis manifests rapidly, with robust cell death within ∼24 h, faster than canonical apoptosis or ferroptosis [22], thereby strengthening both therapeutic impact and scheduling flexibility. Collectively, GOx + cystine + hMnO_2_ constitutes a new and effective combination strategy, distinct from prior pairings of GOx with photodynamic therapy or generic ROS generators [41,42]. Looking ahead, an important avenue is to address alternative NADPH sources—such as glutamine and lactate metabolism—that may sustain redox homeostasis. Combining GOx with inhibitors of these complementary metabolic pathways to jointly suppress NADPH production could further enhance therapeutic efficacy [29,30].

To realize tumor-selective accumulation of GOx and cystine, we engineered a CD24-targeted nanovesicle platform (Cys-hMnO_2_@GOx@EM-CD24). CD24 is a small, highly glycosylated GPI-anchored protein involved in immune cell differentiation and inflammation; when overexpressed on cancer cells, it functions as a “don't-eat-me” signal via Siglec-10, suppressing macrophage and NK-cell clearance [13,43]. CD24 is therefore a promising therapeutic target; our previous work with CD24-targeted ADCs supports its druggability [18]. Here, we confirm CD24 overexpression in neuroblastoma and explore its functional role: CD24 knockdown partially suppresses malignant behavior, but directly targeting CD24 for inhibition alone may be suboptimal, as knockdown effects are modest. By contrast, leveraging high membrane CD24 to mediate antibody-guided endocytosis offers stronger translational potential: CD24 serves as a docking/uptake handle for drug delivery, enabling the nanovesicles to act through binding-and-internalization without relying on epitope-specific inhibitory signaling. On this basis, we provide first-in-model evidence that Cys-hMnO_2_@GOx@EM-CD24 delivered superior antitumor activity with reduced side effects.

Nevertheless, because CD24 is physiologically upregulated in developing neural tissue and neuroblastoma arises in early childhood [20], neurotoxicity warrants careful monitoring. Although NGF-differentiated PC12 neuron-like cells exhibit lower CD24 than tumor cells and reduced sensitivity to Cys-hMnO_2_@GOx@EM-CD24, neuro-safety still requires further validation, particularly in primary sympathetic neuron models and in juvenile mice. Age-stratified safety evaluation of candidate drugs and nanocarriers is also essential.

Finally, a key limitation is our reliance on immunodeficient mouse models, which restricts analysis of nanomedicine–immune microenvironment interactions. Building on our prior hypothesis that disulfidptosis may synergize with immunotherapy [30], and recognizing that partial CD24 blockade could modulate myeloid/NK compartments [43], future studies will test combinations of our platform with immune checkpoint inhibitors, T-cell engagers, or macrophage modulators to potentiate antitumor immunity. Given that metabolic vulnerabilities surrounding NADPH and cystine are conserved across multiple cancer types, we anticipate broader applicability of this strategy to solid tumors beyond neuroblastoma.

## 3 Materials and methods

### 3.1 Cell culture

The human neuroblastoma cell lines SH-SY5Y, SK-N-SH, and SK-N-BE2 (Cell Bank of the Chinese Academy of Sciences, China), SK-N-DZ (ATCC, Manassas, VA, USA), HEK293T embryonic kidney cells, and HK2 human renal tubular epithelial cells (Cell Bank of the Chinese Academy of Sciences, China) were cultured under standard conditions. All cell lines, with the exception of HK2, were maintained in high-glucose Dulbecco's Modified Eagle Medium (DMEM; Procell Life Science & Technology Co., Ltd., Wuhan, China) supplemented with 10 % fetal bovine serum (FBS; Hycezmbio Biotechnology Co., Ltd., Wuhan, China). HK2 cells were cultured in Ham's F-12K (Kaighn's modification) medium (Procell Life Science & Technology Co., Ltd.) with identical supplementation. All cultures were incubated at 37 °C in a humidified 5 % CO_2_ atmosphere.

### 3.2 Reagents and materials

Dialyzed fetal bovine serum (glucose-free) was purchased from Hycezmbio Biotechnology Co., Ltd. (Wuhan, China). Glucose- or cystine-deficient culture media were custom-formulated by Procell Life Science & Technology Co., Ltd. (Wuhan, China). L-Cystine (HY-N0394) and glucose oxidase (HY-P2902) were obtained from MedChemExpress (Monmouth Junction, NJ, USA). Manganese dioxide (MnO_2_), silicon dioxide (SiO_2_), and polyethyleneimine (PEI) were sourced from Macklin Reagent Co., Ltd. (Shanghai, China). Primary antibodies for Western blotting included: FLNA (Cat# A3738; ABclonal Technology, Wuhan, China), MYH9 (Cat# A0173; ABclonal Technology), Drebrin (Cat# 10260-1-AP; Proteintech Group, Wuhan, China), SLC7A11 (Cat# 12691; Cell Signaling Technology [CST], Danvers, MA, USA), Non-reducing protein loading buffer (5X) was acquired from Beyotime Biotechnology Co., Ltd. (Shanghai, China). The NADP+/NADPH Colorimetric Assay Kit (WST-8 method) was procured from Elabscience Biotechnology Co., Ltd. (Wuhan, China).

### 3.3 Cell viability assay

Cell viability was assessed using the Cell Counting Kit-8 (CCK-8; Cat# K1018, APExBIO Technology LLC, Houston, TX, USA). Briefly, cells were seeded into 96-well plates, treated as indicated, and incubated with 100 μL of fresh DMEM containing 10 % CCK-8 reagent for 2 h at 37 °C. Absorbance was measured at 450 nm using a Multiskan GO microplate reader (Thermo Fisher Scientific, Wilmington, DE, USA). To mitigate interference from residual glucose oxidase (GOx) activity, which artificially elevates CCK-8 formazan production, GOx-treated cells underwent three PBS washes followed by 1 h incubation in GOx-free medium prior to CCK-8 assay.

### 3.4 Reducing and non-reducing western blotting

The experiments were performed as described in Gan et al.'s publication [22]. Briefly, cells were lysed in NP-40 buffer containing protease inhibitors. Lysates were sonicated and centrifuged at 12,000×*g* for 15 min at 4 °C. Supernatants were collected, and protein concentrations were determined using a BCA assay (Thermo Fisher Scientific). For reducing conditions, samples were mixed with 5 × Laemmli buffer and denatured at 95 °C for 10 min. For non-reducing conditions, aliquots were combined with non-reducing buffer. Equal protein amounts (20 μg/lane) were resolved on 10 % SDS-PAGE gels and transferred to PVDF membranes (Millipore). Membranes were blocked with RapidBlock™ buffer (ABclonal) for 10 min, incubated with primary antibodies (4 °C, overnight), and probed with HRP-conjugated secondary antibodies (room temperature, 1 h). Signals were detected using ECL Prime (Cytiva). To prevent artifactual oxidation, non-reducing samples were aliquoted, flash-frozen in liquid nitrogen, and stored at −80 °C until use.

### 3.5 NADPH quantification

The assay was performed according to the manufacturer's instructions (NADP+/NADPH Colorimetric Assay Kit, Elabscience Biotechnology Co., Ltd., Wuhan, China). Briefly, cells were lysed in ice-cold extraction buffer. Supernatants were divided into two aliquots:a.Total NADPH/NADP+: Directly analyzed via WST-8 colorimetry. B. NADPH-Specific: Heat-treated at 60 °C for 30 min to degrade NADP + while preserving NADPH. NADPH reduces WST-8 to formazan, with absorbance measured at 450 nm using a microplate reader (Thermo Fisher Scientific, Wilmington, DE, USA).

### 3.6 Cellular uptake assay

To evaluate the tumor-targeting capability of EM-CD24, glucose oxidase (GOx) was fluorescently labeled with FITC and incorporated into Cys-hMnO_2_@GOx@EM-CD24. EM-CD24 or EM were labeled using the PKH26 Red Fluorescent Cell Linker Kit (Sigma-Aldrich, St. Louis, MO, USA) following the manufacturer's protocol. SH-SY5Y and SK-N-DZ cells (20,000 cells/well) were seeded onto 24-well chamber slides and cultured for 24 h in complete medium. Cells were treated with either Cys-hMnO_2_@GOx@EM-CD24 or non-targeted Cys-hMnO_2_@GOx@EM for 6 h, followed by three gentle PBS washes to remove uninternalized nanoparticles. Cells were fixed with 4 % paraformaldehyde (15 min, RT), and nuclei were counterstained with DAPI (1 μg/mL, 10 min). Slides were mounted with antifade medium and air-dried overnight in the dark. Finally, the distribution of EM was observed using a fluorescence microscope.

### 3.7 Cytoskeletal staining

Cytoskeletal architecture was visualized using rhodamine-phalloidin (Cat#16002, ZEN-BIOSCIENCE, Chengdu, China). Cells were seeded on glass coverslips in 24-well plates and subjected to experimental treatments. Following interventions, cells were fixed with 4 % paraformaldehyde (PFA) in PBS (pH 7.4) for 15 min at room temperature (RT), permeabilized with 0.5 % Triton X-100 in PBS for 10 min (RT), and blocked with 0.5 % BSA for 30 min. For F-actin staining, rhodamine-phalloidin working solution (1:50 dilution in PBS) was applied for 20 min at RT. Nuclei were counterstained with DAPI for 10 min. Slides were mounted with antifade medium and air-dried overnight in the dark. Imaging was performed using a Nikon Eclipse Ti2 fluorescence microscope (Tokyo, Japan).

### 3.8 Lentiviral transduction

The anti-CD24 single-chain variable fragment (scFv) sequence was obtained from a patented construct and cloned into the HA-VHH(CD24 scFv)-Myc-GPI lentiviral vector by Shanghai GeneChem Co., Ltd [44,45]. HEK293T cells were transduced with lentiviral particles at an MOI of 10 in the presence of 8 μg/mL Polybrene. Following transduction, cells were initially screened with 2 μg/mL puromycin for 72 h, followed by maintenance in 1 μg/mL puromycin. After 2–3 passages under selection pressure with no significant cell death observed, stable polyclonal populations were expanded. Cells were trypsinized, pelleted, and resuspended in PBS, and GFP-positive cells were purified using fluorescence-activated cell sorting (FACS) based on the viral vector's GFP tag. Validation of successful transduction was performed using RT-PCR and Western blot. Total RNA was extracted, reverse-transcribed, and amplified with scFv-specific primers (Forward: 5′-CTGAGCCTGAGCGTGACCATTG-3′; Reverse: 5′-GGTAGGTCTTTCCATCGCTGTGC-3′), synthesized by Sangon Biotech. Western blot analysis confirmed expression of the HA-tagged (37 kDa) using anti-HA antibodies, with β-actin as a loading control.

### 3.9 Engineered exosome mimics (EM) and exosome preparation

EM was prepared by referring to the method in the previous literature. Brieffy, cells were harvested by scraping and resuspended in PBS. The cell suspension was sequentially extruded through polycarbonate membrane filters (Whatman, Maidstone, UK) with decreasing pore sizes (10 μm → 5 μm → 1 μm) using a mini-extruder (Avanti Polar Lipids, Inc., Alabaster, AL, USA), with three passes performed for each pore size. The resulting extrudate was subjected to ultracentrifugation (100,000 g, 70 min, 4 °C), filtered through a 0.22 μm sterile membrane filter (Millipore), and stored at −80 °C in single-use aliquots to minimize freeze-thaw cycles. Exosome were isolated from HEK293T cell culture supernatants using differential centrifugation. Briefly, conditioned media were sequentially centrifuged at 300 g for 10 min, 2000 g for 10 min, and 10,000 g for 30 min (4 °C) to remove cellular debris and apoptotic bodies. Clarified supernatants were then ultracentrifuged at 100,000 gfor 70 min (4 °C; Beckman Optima L80XP ultracentrifuge, Beckman Coulter, Brea, CA, USA). The resultant exosome were resuspended in PBS and subjected to a final ultracentrifugation wash (100,000×*g*, 70 min, 4 °C). Purified EVs were filtered through a 0.22 μm sterile membrane, aliquoted, and stored at −80 °C to minimize freeze-thaw cycles.

### 3.10 Synthesis of Cys-hMnO2@GOx@EM-CD24 nanocomposite

The nanocomposite was synthesized through a multi-step hierarchical fabrication process. Initially, 0.4 g cetyltrimethylammonium bromide (CTAB) and 1000 mg L-cystine were dissolved in 10 mL deionized water, with the pH adjusted to 9.5. After adding 100 μL triethylamine, the mixture was magnetically stirred at 95 °C for 30 min, followed by the dropwise addition of 1.2 mL tetraethyl orthosilicate (TEOS) under continuous stirring for 4 h. After collection via centrifugation, the Cys-hSiO_2_ nanoparticles underwent triple washing with ethanol and subsequently with deionized water. The purified product was finally resuspended and adjusted to a total volume of 50 ml using deionized water. Then 50 mg of potassium permanganate (KMnO4) was introduced into the above suspension. The mixture was then subjected to ultrasonication (40 kHz, 120 W) for 1 h followed by continuous magnetic stirring at 1200 rpm for 6 h. The product was then treated with 24 mL of 2 mol/L sodium carbonate solution under reflux at 60 °C for 3 h, followed by a second reflux step in 3 % sodium bicarbonate solution for 10 h to etch the silica template, yielding hollow MnO2 nanostructures loaded with cystine (Cys-hMnO_2_). The particles were washed with deionized water until the supernatant reached pH 7 (tested by pH strips). After the final wash, the pellet was resuspended in deionized water and volumetrically adjusted to 5 ml. This nanoparticle suspension was then incubated with 5.0 ml polyethyleneimine solution (PEI, MW 10000, 1 mg•ml^−1^) under magnetic stirring (500 rpm) for 2 h.The nanoparticles were purified by centrifugal washing and resuspended in deionized water to a final volume of 10 ml, yielding PEI-functionalized Cys-hMnO2 (Cys-MnO2-PEI). This dispersion was then incubated with 20 mg GOx under shaking (500 rpm, 60min), purified again via centrifugal washing, and volumetrically adjusted to 10 ml to yield the Cys-hMnO_2_@GOx.

For EM or EM-CD24 coating, 0.3 ml Cys-MnO_2_@GOx was mixed with 0.3 mL CD24-engineered exosome (EM-CD24) or plain exosome (EM) solution (4 mg/ml). The mixture underwent sequential extrusion through a polycarbonate membrane (400 nm, 20 passes) followed by a lipid extruder (200 nm, 10 passes). Final products were centrifuged and resuspended in 0.6 mL PBS. Control formulations (hMnO2@GOx@EM-CD24 without cystine, Cys-hMnO_2_@EM-CD24 without GOx) were prepared using identical protocols with omitted components.

### 3.11 Particle size and zeta potential analysis

The hydrodynamic diameters and zeta potentials of the nanocomposites (hMnO2@GOx@EM-CD24, Cys-hMnO2@EM-CD24, Cys-hMnO2@GOx@EM-CD24, and Cys-hMnO2@GOx@EM) were measured using a Brookhaven Instruments analyzer.

### 3.12 HPLC analysis

Nanocomposite samples (Cys-hMnO2@EM-CD24, Cys-hMnO2@GOx@EM-CD24, and Cys-hMnO2@GOx@EM) were diluted 10-fold in deionized water. A 0.2 mL aliquot of each sample was mixed with 1.4 mL of 4 mg/mL Fmoc-OSu solution and incubated at 40 °C for 1 h. After centrifugation (12,000×*g*, 5 min), the supernatant was analyzed by HPLC (Agilent 1260) equipped with a ZORBAX SB-C18 column (4.6 × 250 mm, 5 μm). The mobile phase consisted of methanol:water (70:30 v/v) at 0.7 mL/min, with column temperature maintained at 35 °C. Detection wavelength was set to 300 nm, and injection volume was 20 μL. System calibration was performed using Fmoc-L-Cys standards processed identically.

### 3.13 XPS sample preparation

Cys-hMnO2-PEI powder (5–10 mg) was homogenized in an ethanol-cleaned agate mortar by unidirectional grinding for 15–20 min to achieve a uniform fine texture. The powder was transferred to a pellet die, pre-pressed at 1 MPa for 30 s to remove air pockets, and then compressed at 8–10 MPa for 2 min to form a dense, cohesive pellet. The pellet was dried in a vacuum oven at 25 °C and 0.1 mbar for 2 h to desorb surface moisture. The dried pellet was mounted on an XPS holder using conductive carbon tape, ensuring electrical contact without sputter coating to avoid elemental interference.

### 3.14 L-cystine release profiling

Three nanocomposite formulations (Cys-hMnO_2_@EM-CD24, Cys-hMnO_2_@GOx@EM-CD24, Cys-hMnO_2_@GOx@EM) were evaluated for cystine release kinetics using dialysis membrane methodology. For each formulation, 1 mL of sample was loaded into a 500 Da molecular weight cutoff (MWCO) dialysis bag, securely sealed, and immersed in 20 mL of release medium (0.01 M PBS, pH 7.4, containing 0.1 % Tween 80 and 5.5 mM glucose). The system was maintained at 37 °C under continuous magnetic stirring (100 rpm). Aliquots (50 μL) were collected at predetermined intervals (0 min, 10 min, 30 min, 1 h, 2 h, 4 h, 8 h, 12 h, 24 h, and 48 h), with the medium replenished with an equal volume of fresh buffer after each sampling. Cystine quantification was performed via HPLC (Section 2.3) to determine cumulative release profiles. The cumulative drug release amount was calculated according to the following formula.

$$E_{r}=\left(V_{e}\sum_{1}^{n-1}C_{i}+V_{0}C_{n}\right)/m_{drug}$$

($E_{r}$:Cumulative drug release amount; $V_{e}$:Volume of PBS replaced; $V_{0}$:

Total volume of release medium; $C_{i}$:Drug concentration in the release medium at the i-th sampling point; $m_{drug}$:Total mass of loaded drug;*n*:Number of PBS replacements).

### 3.15 Oxygen generation profiling

The oxygen production kinetics of three nanocomposite formulations (hMnO_2_@GOx@EM-CD24, Cys-hMnO_2_@GOx@EM-CD24, Cys-hMnO_2_@GOx@EM were quantified using a JPSJ-605F dissolved oxygen meter (Shanghai Yitian Scientific Instrument Co., Ltd.). For each sample, 2 mL of nanocomposite suspension was loaded into dialysis bags (500 Da MWCO) and immersed in 20 mL of medium (0.01 M PBS, pH 7.4, containing 0.1 % Tween 80 and 5.5 mM glucose). The dissolved oxygen probe was positioned at a fixed depth within the medium under constant magnetic stirring (100 rpm) at 37 °C. Real-time oxygen concentration (% saturation) was recorded at predetermined intervals (0 h, 0.25 h, 0.5 h, 2 h, 6 h, 12 h, 24 h, 36 h, 48 h), with triplicate measurements per timepoint. Prior to each reading, the system was equilibrated for 30 s to stabilize probe signals.

### 3.16 TEM morphology and EDS mapping

Polyethylenimine (PEI)-modified Cys-hMnO_2_ (Cys-hMnO_2_-PEI) was dispersed in deionized water (0.1 mg/mL), sonicated for 15 min, and deposited onto ethanol-cleaned ultrathin carbon-coated copper grids (300 mesh). Following 90 s of adsorption, excess liquid was removed using filter paper, and samples were desiccated for 2 h. Elemental mapping (C, N, S, O, Si, Mn) and morphological analysis were performed using an FEI Talos F200S transmission electron microscope operated at 200 kV.

hMnO_2_@GOx@EM-CD24, or Cys-hMnO_2_@EM-CD24, Cys-hMnO_2_@GOx@EM-CD24, Cys-hMnO_2_@GOx@EM were deposited onto pre-treated transmission electron microscopy grids. Following 60 s of adsorption, the samples were negatively stained with 2.5 % phosphotungstic acid (pH 6.8) and imaged using a transmission electron microscope operated at 80 kV.

### 3.17 Animal studies

Male NOD/SCID mice (6 weeks old) were housed in specific pathogen-free facilities under controlled conditions following protocols approved by the Animal Ethics Committee of Children's Hospital Affiliated to Chongqing Medical University (IACUC Issue No: CHCMU-IACUC20220323001). For the orthotopic neuroblastoma model, luciferase-transfected SK-N-DZ cells (2 ∗10^6^ cells in 50 μL medium) were surgically implanted into the left adrenal gland. Tumor engraftment was confirmed by bioluminescence imaging (BLI) on day 10, and mice were randomized into six groups: PBS (vehicle control, group 1), hMnO_2_@EM-CD24 (empty vesicles, group 2), Cys-hMnO_2_@EM-CD24 (cystine monotherapy, group 3), hMnO_2_@GOx@EM-CD24 (glucose oxidase monotherapy, group 4), Cys-hMnO_2_@GOx@EM-CD24 (combination therapy, group 5), and Cys-hMnO_2_@GOx@EM (non-targeted control, group 6). Treatments were administered via tail vein every 48 h until study termination (40 mg/kg). Tumor progression was monitored using an in vivo imaging system, and mouse body weight was recorded. For the metastatic lung model, SK-N-DZ cells (1∗10^6^ cells in 100 μL PBS) were injected into the tail vein of NOD/SCID mice, and lung metastasis was confirmed by BLI on day 14 before initiating treatment protocols. Subsequently, the drug was administered and the changes in luminescence intensity were continuously detected. At study endpoint, mice were euthanized by CO_2_ asphyxiation. Tumors and major organs (heart, liver, spleen, kidneys, lungs) were harvested, weighed, and fixed in 10 % neutral buffered formalin for histopathological analysis. Serum biochemical parameters (ALT, AST, creatinine, CK-MB, urea) were analyzed by the Clinical Laboratory of Chongqing Children's Hospital. Paraffin-embedded tissues were sectioned and stained with hematoxylin and eosin (H&E) to assess tumor morphology. Similarly, another group of mice was modeled and intervened, but not sacrificed, and their survival conditions were observed. For compared with the clinically used agent doxorubicin, doxorubicin was given by tail-vein injection (intravenous) at 2.5 mg/kg on days 10, 12, 14, 16 and 18, yielding a cumulative dose of 12.5 mg/kg; mice were harvested on day 20, with body weight monitored throughout. All procedures adhered to NIH guidelines and local regulatory standards for animal welfare.

### 3.18 In vivo targeting specificity assessment

To evaluate tumor-selective biodistribution, DiR-labeled Cys-hMnO_2_@GOx@EM-CD24 (dEM-CD24), non-targeted Cys-hMnO_2_@GOx@EM (dEM), or free DiR were intravenously administered to orthotopic and metastatic model mice on days 10 or 14 post-tumor implantation. Whole-body fluorescence was longitudinally monitored using an IVIS Spectrum imaging system at 6, 12, 24, 48, 72, and 96 h post-injection. Given that fluorescence intensity peaked at 24 h post-injection, a cohort of mice was euthanized at this time point, and major organs (heart, liver, spleen, lungs, and kidneys) alongside tumors were harvested for ex vivo fluorescence quantification. Radiant efficiency was analyzed using Living Image software. For cellular-resolution distribution analysis, a parallel cohort received DiO-labeled or DiL-labeled vesicles. Harvested tissues were cryosectioned (10 μm), counterstained with DAPI (1 μg/mL, 10 min).

### 3.19 Immunofluorescence (IF), immunohistochemistry (IHC), H&E staining, and cryosectioning

Tissue specimens were fixed in 4 % paraformaldehyde, paraffin-embedded, and sectioned into 4 μm slices. For antigen retrieval, deparaffinized sections were heated in citrate buffer (pH 6.0) at 95 °C for 15 min and blocked with 0.5 % bovine serum albumin (BSA) for 1 h at room temperature. Primary antibodies (PCNA, MMP2; diluted 1:200 in 0.5 % BSA) were applied and incubated at 4 °C overnight. After three PBS washes, fluorophore-conjugated secondary antibodies (1:200 in PBS) were incubated for 1 h at room temperature. Nuclei were counterstained with Dapi, and slides were imaged using a fluorescence microscope. For IHC, a similar protocol was followed using primary antibodies against SLC7A11、CD24 (1:200 in 0.5 % BSA). After secondary antibody incubation (HRP-conjugated, 1:200), signals were developed with 3,3′-diaminobenzidine (DAB) substrate and counterstained with hematoxylin. H&E staining was performed by sequential deparaffinization, rehydration, hematoxylin incubation (5 min), eosin counterstaining (1 min), and dehydration through graded ethanol/xylene solutions. Frozen sections were prepared by embedding tissue in OCT compound. The sections were mounted onto glass slides, air-dried briefly. Finally, the slides were counterstained (with DAPI), mounted with antifade reagent, and imaged under a fluorescence or confocal microscope.

### 3.20 Statistical analysis

Statistical analyses were performed in GraphPad Prism v8.0.2 and R v4.3.1. Dose–response data were fitted with a 4-parameter logistic (4 PL) model to estimate IC50 with 95 % CIs. For multi-group comparisons, one-way ANOVA with Tukey's post-hoc test was used; when normality (Shapiro–Wilk) or homoscedasticity (Levene) was violated, Welch's ANOVA with Games–Howell post-hoc or the Kruskal–Wallis test with Dunn's correction was applied. Time-course experiments were analyzed by two-way ANOVA; if the same wells were followed over time, a repeated-measures model with Greenhouse–Geisser correction was used. Two-group contrasts used (Welch's) two-tailed Student's t-tests. Survival curves were generated with the survival package and visualized with survminer; overall survival was compared by the log-rank test (α = 0.05). Unless otherwise stated, n denotes independent biological replicates; for animal studies, individual animals. Technical replicates were averaged and are not counted in n. Statistical significance: ns represents P > 0.05, ∗ represents P < 0.05, ∗∗ represents P < 0.01, ∗∗∗ represents P < 0.001.

## CRediT authorship contribution statement

**Tao Mi:** Writing – review & editing, Writing – original draft, Visualization, Software, Resources, Methodology, Formal analysis, Data curation, Conceptualization. **Junhong Liu:** Software, Data curation. **Junyi Luo:** Software, Methodology, Data curation. **Xiangpan Kong:** Investigation, Data curation. **XiaoJun Tan:** Funding acquisition, Data curation. **Liming Jin:** Software, Investigation, Formal analysis. **Peng Guo:** Writing – review & editing, Data curation. **Dawei He:** Writing – review & editing, Validation, Supervision, Project administration, Funding acquisition, Conceptualization.

## Ethics approval and consent to participate

This retrospective study involving human participants was in accordance with the ethical standards of the Ethics Committee of Children's Hospital affiliated to Chongqing Medical University. All participants provided written informed consent. All animal procedures were approved according to the guidelines of the Animal Ethics Committee of the Children's Hospital of Chongqing Medical University (CHCMU-CHCMU-IACUC20220323001).

## Funding

The Chongqing Traditional Chinese Medicine Innovation Team: “Innovative Team for the Development of New Targeted Delivery Traditional Chinese Medicine Formulations.” Chongqing medical scientific research project (joint project of Chongqing Health Commission and Science and Technology Bureau) (2025ZDXM036). Project of Chongqing Natural Science Foundation (CSTB2024NSCQ-MSX0270). 10.13039/501100020207Health Commission of Sichuan Province Medical Science and Technology Program (24QNMP084).

## Declaration of competing interest

The authors declare that they have no competing interests.

## Data Availability

Data will be made available on request.
