# Supplementary material for: CD24-targeted cystine and glucose oxidase cascade catalytic nanosystem triggers disulfidptosis in neuroblastoma
Source: Mater Today Bio. 2025 Nov 1;35:102496. doi: 10.1016/j.mtbio.2025.102496 (PMC12639498; doi:10.1016/j.mtbio.2025.102496)
Supplement: Multimedia component 1 [file mmc1.docx]

**CD24-Targeted glucose oxidase and cystine cascade catalytic nanosystem Trigger Disulfidptosis in Neuroblastoma**

Tao Mi^1^, Junhong Liu^1^, Junyi Luo^1^, Xiangpan Kong^1^, XiaoJun Tan^1,3^, Liming Jin^1^, Peng Guo^1,4^, Dawei He^1,2^*

1. Department of Urology, Children's Hospital of Chongqing Medical University, National Clinical Research Center for Child Health and Disorders, Ministry of Education Key Laboratory of Child Development and Disorders, Children Urogenital Development and Tissue Engineering of Chongqing Education Commission of China, the Laboratory of Targeted Delivery of Traditional Chinese Medicine.
2. The Second Affiliated Hospital of Chongqing Medical University, Key Laboratory of Integrated Therapy of Traditional Chinese Medicine for Tumors, Chongqing Municipal Administration of Traditional Chinese Medicine.
3. Beijing Anzhen Nanchong Hospita, Capital Medical University & Nanchong Central Hospital. The Second Clinical Medical College of North Sichuan Medical College.
4. Institute of Basic Medicine and Cancer (IBMC), Chinese Academy of Sciences, Hangzhou, Zhejiang, 310022 China.

**Supplementary Materials**

**Methods:**

**1.1. Reagents and Materials**

Dialyzed fetal bovine serum (glucose-free) was purchased from Hycezmbio Biotechnology Co., Ltd. (Wuhan, China). Glucose- or cystine-deficient culture media were custom-formulated by Procell Life Science & Technology Co., Ltd. (Wuhan, China). L-Cystine (HY-N0394) and glucose oxidase (HY-P2902) were obtained from MedChemExpress (Monmouth Junction, NJ, USA). Manganese dioxide (MnO_2_), silicon dioxide (SiO_2_), and polyethyleneimine (PEI) were sourced from Macklin Reagent Co., Ltd. (Shanghai, China). Primary antibodies for Western blotting included: FLNA (Cat# A3738; ABclonal Technology, Wuhan, China), MYH9 (Cat# A0173; ABclonal Technology), Drebrin (Cat# 10260-1-AP; Proteintech Group, Wuhan, China), SLC7A11 (Cat# 12691; Cell Signaling Technology [CST], Danvers, MA, USA), Non-reducing protein loading buffer (5X) was acquired from Beyotime Biotechnology Co., Ltd. (Shanghai, China). The NADP+/NADPH Colorimetric Assay Kit (WST-8 method) was procured from Elabscience Biotechnology Co., Ltd. (Wuhan, China). Intracellular ROS were quantified using a commercial kit (Cat. MA0219; Dalian Meilun Biotechnology Co., Ltd., Dalian, China). Lipid peroxidation was assessed with an MDA/TBARS assay kit (Cat. WLA048a; Beyotime Biotechnology, Shanghai, China). GSH and oxidized glutathione (GSSG) were measured with an enzymatic recycling GSH/GSSG assay kit (Cat. S0053; Beyotime Biotechnology, Shanghai, China). All kits were used strictly according to the manufacturers’ instructions.

**1.2. Cell Viability Assay**

Cell viability was assessed using the Cell Counting Kit-8 (CCK-8; Cat# K1018, APExBIO Technology LLC, Houston, TX, USA). Briefly, cells were seeded into 96-well plates, treated as indicated, and incubated with 100 μL of fresh DMEM containing 10% CCK-8 reagent for 2 h at 37°C. Absorbance was measured at 450 nm using a Multiskan GO microplate reader (Thermo Fisher Scientific, Wilmington, DE, USA).To mitigate interference from residual glucose oxidase (GOx) activity, which artificially elevates CCK-8 formazan production, GOx-treated cells underwent three PBS washes followed by 1 h incubation in GOx-free medium prior to CCK-8 assay.

**1.3. Reducing and Non-Reducing Western Blotting**

The experiments were performed as described in Gan et al.'s publication[1]. Briefly, cells were lysed in NP-40 buffer containing protease inhibitors. Lysates were sonicated and centrifuged at 12,000 × g for 15 min at 4°C. Supernatants were collected, and protein concentrations were determined using a BCA assay (Thermo Fisher Scientific). For reducing conditions, samples were mixed with 5× Laemmli buffer and denatured at 95°C for 10 min. For non-reducing conditions, aliquots were combined with non-reducing buffer. Equal protein amounts (20 μg/lane) were resolved on 10% SDS-PAGE gels and transferred to PVDF membranes (Millipore). Membranes were blocked with RapidBlock™ buffer (ABclonal) for 10 min, incubated with primary antibodies (4°C, overnight), and probed with HRP-conjugated secondary antibodies (room temperature, 1 h). Signals were detected using ECL Prime (Cytiva).To prevent artifactual oxidation, non-reducing samples were aliquoted, flash-frozen in liquid nitrogen, and stored at -80°C until use.

**1.4. Stability studies of the nanoparticles and Quantification of GOx Enzymatic Activity**

Nanocomposite suspensions (hMnO_2_@GOx@EM-CD24, Cys-hMnO_2_@EM-CD24, Cys-hMnO_2_@GOx@EM-CD24, Cys-hMnO_2_@GOx@EM) were diluted to 1 mg/mL in PBS (pH 7.4) containing 10% FBS. Samples (2 mL each) were incubated at 37°C with gentle orbital shaking (100 rpm) to simulate mild shear forces without inducing artificial disruption. Hydrodynamic diameter and zeta potential were measured using a Brookhaven Instruments analyzer at time points: 0 h, 12h, 24 h, and 48 h. Aliquots (0.5 mL) were withdrawn and measured. To detect the ability of the material to generate H₂O₂.Nanocomposite suspensions (2 mL at 1 mg/mL for MnO₂@GOx@EM-CD24, Cys-hMnO₂@GOx@EM-CD24, and Cys-hMnO₂@GOx@EM) were loaded into dialysis bags (500 Da MWCO) and immersed in 20 mL of medium (0.01 M PBS, pH 7.4, containing 0.1% Tween 80 and 5.5 mM glucose) at 37°C under magnetic stirring (100 rpm). This setup mirrors the oxygen generation profiling (Section 3.16) to ensure comparability. Aliquots (100 μL) from the external medium were collected at predetermined intervals (0 h, 0.25 h, 0.5 h, 2 h, 6 h, 12 h, 24 h, 36 h, 48 h), mixed with 100 μL 5 μg/mL HRP, 100 μL 0.5 mg/mL ABTS, and 700 μL deionized water (total 1 mL reaction volume), and incubated for 5 min to develop color. Absorbance was measured at 414 nm using a spectrophotometer. H2O2 concentrations were interpolated from a standard curve established with final H2O2 concentrations of 0, 0.5, 2, 5, and 10 μM. Cys-hMnO₂@EM-CD24 (without GOx) served as a negative control, yielding negligible H2O2 (<0.1μM). Measurements were performed in triplicate, with medium replenished post-sampling to maintain volume.

**1.5. NADPH Quantification**

The assay was performed according to the manufacturer’s instructions (NADP+/NADPH Colorimetric Assay Kit, Elabscience Biotechnology Co., Ltd., Wuhan, China). Briefly, cells were lysed in ice-cold extraction buffer. Supernatants were divided into two aliquots:a.Total NADPH/NADP+: Directly analyzed via WST-8 colorimetry. B.NADPH-Specific: Heat-treated at 60°C for 30 min to degrade NADP+ while preserving NADPH. NADPH reduces WST-8 to formazan, with absorbance measured at 450 nm using a microplate reader (Thermo Fisher Scientific, Wilmington, DE, USA).

### **1.6. Malondialdehyde (MDA) assay**

MDA was quantified with a TBA-reactive substances kit (Beyotime, Cat. WLA048a) in neuroblastoma cells and mouse liver/kidney processed on ice. Cells were rinsed in PBS and lysed; tissues (~10–30 mg) were homogenized in PBS or Beyotime lysis buffer (P0013) at ~10% w/v (cells: ~0.1 mL buffer per 1 × 10⁶ cells), and supernatants were clarified (10,000–12,000 g, 10 min, 4 °C). Aliquots (0.1 mL) of samples, blanks (PBS/lysis buffer), and MDA standards (processed in parallel) were mixed with 0.2 mL freshly prepared MDA working solution, heated at 100 °C for 15 min, cooled, centrifuged (1,000 g, 10 min), and the supernatant (200 µL) was read at 532 nm (reference 450 nm optional). Concentrations were interpolated from the standard curve; cellular/tissue results were normalized to protein (BCA) and reported as µmol/mg protein (or per g wet tissue as specified). To determine the change trend, normalized MDA levels were expressed as fold-change of the control (with reagent blanks and matrix-matched controls included to correct background and potential nanomaterial interference).

### **1.7. Glutathione (GSH) assay**

GSH and GSSG were quantified with a commercial kit (Beyotime, Cat. S0053) in neuroblastoma cells treated with Cys-hMnO₂@GOx@EM-CD24 and in mouse liver samples harvested at the indicated time points. Cells or 10–30 mg tissue were homogenized/lysed on ice; supernatants were deproteinized with freshly prepared 5% protein-removal reagent M and clarified (10,000 × g, 10 min, 4 °C). Total glutathione (GSH + 2×GSSG) was determined by the DTNB/glutathione-reductase recycling reaction at A412 nm after NADPH addition; GSSG was measured from parallel aliquots pretreated with the kit’s GSH-removal reagents (25 °C, 60 min). Concentrations were interpolated from GSSG standards processed identically, and GSH was calculated as Total − 2×GSSG. Cellular results were normalized to protein (BCA; nmol/mg protein); hepatic/renal results were expressed per mg protein or per g wet tissue as specified. Reagent blanks and matrix-matched controls (including nanomaterial-only controls for cell assays) were included to correct background/interference.

**1.8. Cytoskeletal Staining**

Cytoskeletal architecture was visualized using rhodamine-phalloidin (Cat#16002, ZEN-BIOSCIENCE, Chengdu, China). Cells were seeded on glass coverslips in 24-well plates and subjected to experimental treatments. Following interventions, cells were fixed with 4% paraformaldehyde (PFA) in PBS (pH 7.4) for 15 min at room temperature (RT), permeabilized with 0.5% Triton X-100 in PBS for 10 min (RT), and blocked with 0.5% BSA for 30 min. For F-actin staining, rhodamine-phalloidin working solution (1:50 dilution in PBS) was applied for 20 min at RT. Nuclei were counterstained with DAPI for 10 min. Slides were mounted with antifade medium and air-dried overnight in the dark. Imaging was performed using a Nikon Eclipse Ti2 fluorescence microscope (Tokyo, Japan).

**1.9. Immunofluorescence (IF), Immunohistochemistry (IHC).**

For immunofluorescence staining on cells, tumor cells were seeded on sterile coverslips in 24-well plates and cultured overnight. Cells were fixed with 4% paraformaldehyde for 15 min, and blocked with 5% BSA for 30 min at room temperature. Primary antibodies against CD24 (1:200 in 0.5% BSA) were incubated overnight at 4°C, followed by fluorophore-conjugated secondary antibodies for 1 h at room temperature in the dark. Nuclei were counterstained with DAPI, and coverslips were mounted with antifade reagent for fluorescence imaging. For IHC, Tumor tissues were harvested, fixed in 4% paraformaldehyde overnight, embedded in paraffin, and sectioned at 4 μm thickness.antigen retrieval was performed in citrate buffer at 95°C for 15 min. After cooling, sections were blocked with 5% BSA for 30 min and incubated overnight at 4°C with primary antibodies against SLC7A11 and CD24 (1:200 in 0.5% BSA). After incubation with HRP-conjugated secondary antibodies (1:200), signals were developed using DAB substrate and counterstained with hematoxylin. For IF, a similar protocol was followed using primary antibodies against MMP21、PCNA (1:200 in 0.5% BSA). Then sections/cells were incubated with fluorophore-conjugated secondary antibodies (diluted 1:2000 in PBS) for 1 hour at room temperature. Nuclei were counterstained with DAPI, and slides were imaged using a fluorescence microscope.

**1.10. siRNA transfection**

The siRNAs for CD24, and corresponding negative controls were purchased from Tsingke Biotechnology Co., Ltd. (China). The information of the siRNA is shown in table 1. According to the manufacturer guidelines, we used Lipofectamine 2000 for transfections. The subsequent scratch-wound and Transwell assays were performed according to our previously published protocols[2].

**Table 1. The sequence of the si-RNA**

| Target gene name | sense（5'-3'） | antisense（5'-3'） |
| --- | --- | --- |
| CD24-a | ACGCAGAUUUAUUCCAGUGAA | UUCACUGGAAUAAAUCUGCGU |
| CD24-b | ACUCCUCCCAGAGUACUUCCA | UGGAAGUACUCUGGGAGGAGU |
| CD24-c | CUUCUGCAUCUCUACUCUUAA | UUAAGAGUAGAGAUGCAGAAG |

**1.11. Neurotoxicity Assessment in PC12 cells**

Rat pheochromocytoma PC12 cells (ATCC CRL-1721) were maintained in RPMI-1640 supplemented with 10% horse serum, 5% fetal bovine serum, and 1% penicillin–streptomycin at 37°C, 5% CO2 on collagen/laminin-coated plates. For neuronal differentiation, cells were switched to reduced-serum medium (1% horse, 0.5% FBS) containing NGF (100 ng/mL) for 72 h , with half-medium changes every 24 h. NGF-differentiated PC12 cells were exposed to graded concentrations of Cys-hMnO_2_@GOx@EM-CD24, and cell viability was quantified by the CCK-8 assay.

**1.12.Flow cytometry—Apoptosis (Annexin V/PI).**

Cells were treated as indicated (24 h cystine deprivation or supplementation). Both floating and adherent cells were collected, washed in cold PBS, and stained with Annexin V–FITC and propidium iodide (PI) in binding buffer (10 mM HEPES, 140 mM NaCl, 2.5 mM CaCl₂) for 15 min at room temperature in the dark. Samples were acquired within 1 h on a flow cytometer (e.g., BD LSR/Fortessa). Debris was excluded by FSC/SSC, and doublets were removed by FSC-H vs FSC-A. Quadrants were defined as live (Annexin V⁻/PI⁻), early apoptosis (Annexin V⁺/PI⁻), and late apoptosis/necrosis (Annexin V⁺/PI⁺).

**1.13. Flow cytometry—Cell cycle (PI/RNase).**

Following the indicated treatments, cells were harvested, washed with PBS, and fixed dropwise in ice-cold 70% ethanol at −20 °C (≥2 h or overnight). Fixed cells were washed and incubated with RNase A (100 µg/mL, 30 min, 37 °C), then stained with PI (50 µg/mL) in PBS (optional 0.1% Triton X-100) for 15 min at room temperature in the dark. At least 20,000 events/sample were collected. Doublets were excluded using PI-W/PI-A gating, and phase distribution (G₀/G₁, S, G₂/M) was calculated using Watson Pragmatic or Dean–Jett–Fox models (FlowJo/ModFit LT).

**1.14. Intracellular ROS assay**

After treatments, cells were rinsed twice with pre-warmed phenol-red–free, serum-free culture medium, then incubated with DCFH-DA (10 µM in the same medium; protected from light) for 30 min at 37 °C. Cells were washed three times with phenol-red–free, serum-free medium and immediately recorded by epifluorescence microscopy and by microplate reader (excitation 488 nm, emission 525 nm, FITC channel). Optional nuclear counterstaining was performed with Hoechst 33342 (2 µg/mL, 5 min). Exposure/gain (microscopy) and integration settings (plate reader) were held constant across groups. Background signals from reagent-only medium and nanomaterial-only (no-cell) wells processed in parallel were subtracted prior to analysis. Results were normalized to the vehicle control within each experiment.

1. **Results:**
   1. **CD24 has emerged as a potential therapeutic target in neuroblastoma**

CD24 has emerged as a pivotal therapeutic target in oncology[3]. While our previously engineered CD24-directed antibody-drug conjugate (ADC) demonstrated favorable tumor selectivity, the restricted payload capacity of monoclonal antibodies limited its therapeutic versatility[4]. To enable precise co-delivery of multiple disulfidptosis inducers, we developed anti-CD24 single-chain variable fragments (anti-CD24 scFv)-functionalized extracellular vesicles (EM-CD24) using a engineering strategy. To substantiate CD24's targetability in neuroblastoma, single-cell RNA sequencing data from 16 primary neuroblastoma tumors (GSE137804) were rigorously analyzed. Following stringent quality control, 171,636 cells were subjected to UMAP-based clustering and marker-guided annotation, identifying eight distinct subsets dominated by neurogenic populations (Figure S1A). Transcriptomic profiling revealed significantly elevated CD24 expression in tumor cells versus stromal/immune compartments (Figure S1B-C). Immunohistochemical analysis of 32 clinical specimens confirmed membrane/cytoplasmic CD24 positivity in 87.5% (28/32) of cases, suggesting broad clinical applicability (Figure S1D). Parallel validation in neuroblastoma cell lines (SK-N-DZ/SH-SY5Y) demonstrated that CD24 is highly expressed in these cell lines and predominantly localizes to the membrane (Figure S1E-F).


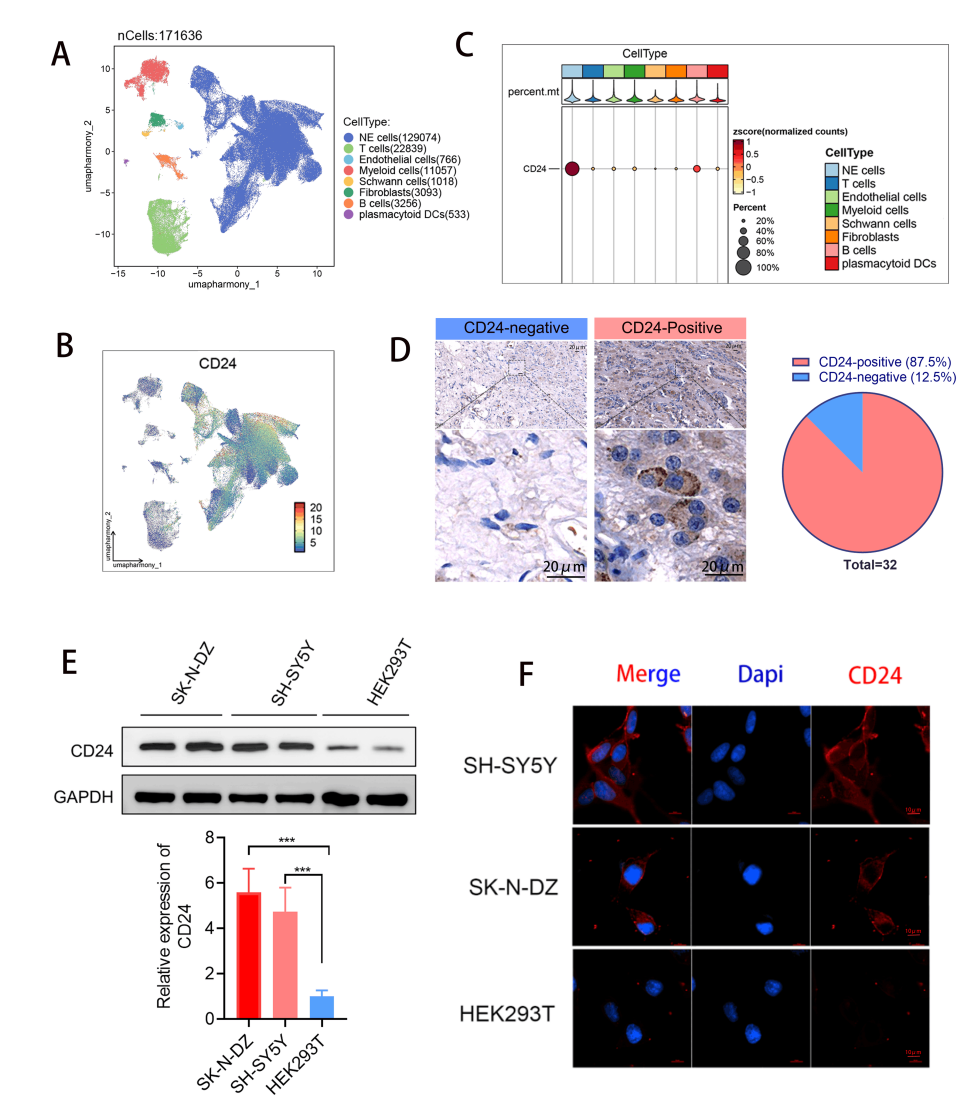


**Figure S1. CD24 is highly expressed in neuroblastoma cells.** (A) Single-cell RNA sequencing analysis of neuroblastoma (GSE137804 dataset) was performed, and cell clustering was visualized using UMAP. (B-C) CD24 expression across different cell clusters. (D) Immunohistochemical validation of CD24 expression in clinical neuroblastoma specimens. (E-F) Western blot (E) ( n=3, mean ± SD) and immunofluorescence (F) confirming CD24 expression in neuroblastoma cell lines. Statistical comparisons by one-way ANOVA with Tukey post hoc tests; *** represents P < 0.001.

- 1. **CD24 knockdown reduces neuroblastoma aggressiveness**

To further substantiate the function of CD24, we conducted loss-of-function assays in SK-N-DZ and SH-SY5Y neuroblastoma cells: three non-overlapping siRNAs efficiently reduced CD24 at the mRNA and protein levels (Figure S2A-B), and two high-efficiency siRNAs were used for phenotyping. CD24 depletion significantly decreased cell proliferation and impaired motility, as evidenced by reduced wound closure in scratch assays and decreased Transwell invasion (Figure S2C-E). Consistently, immunoblotting revealed down-regulation of the invasion-associated protease MMP2 and the proliferation marker PCNA upon CD24 knockdown (Figure S2F-G).The above results demonstrate that CD24 can serve as a therapeutic target for neuroblastoma. We then generated EM-CD24 following the workflow illustrated in Figure S2H.


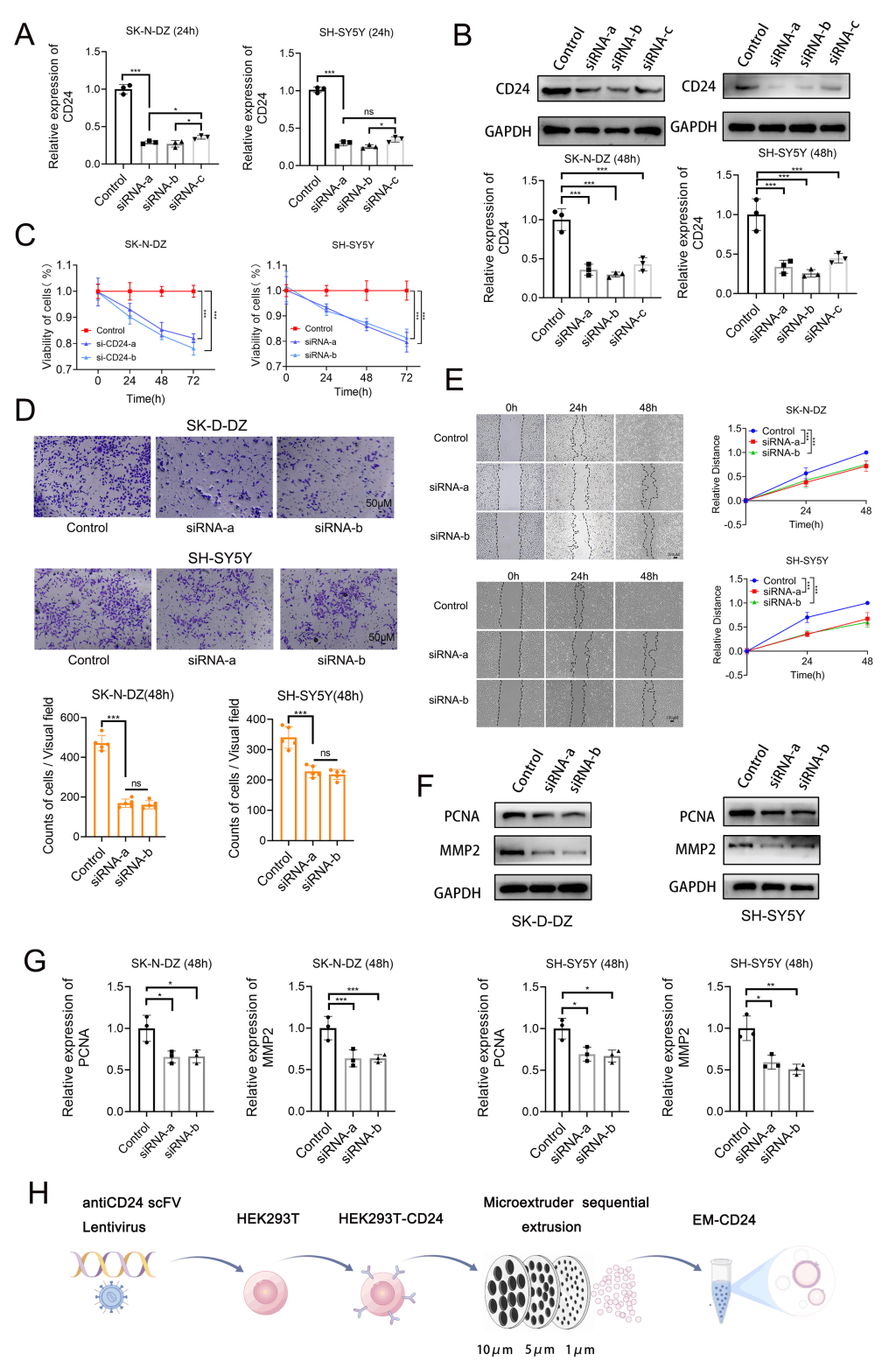


**Figure S2. CD24 knockdown reduces neuroblastoma aggressiveness.** (A) RT-PCR verified that si-CD24 inhibited mRNA expression of CD24 ( n=3, mean ± SD). (B) The siRNA inhibited the protein expression of CD24 ( n=3, mean ± SD). (C) CCK8 detected the inhibitory effect of siRNA on the proliferation of **neuroblastoma** cells( n=5, mean ± SD). (D-E) Down-regulation of CD24 inhibited the invasion and migration of **neuroblastoma** cells ( n=5, mean ± SEM). (F-G) The protein expression levels of MMP2, PCNA ( n=3, mean ± SEM). (H) Schematic of EM-CD24 biofabrication: Lentiviral transduction of HEK293T cells with anti-CD24 single-chain variable fragments (anti-CD24 scFv), followed by extrusion-based nanovesicle generation. Two-way ANOVA with Tukey’s post hoc (C, E). One-way ANOVA with Tukey’s post hoc (A,B,D,G); ns represents P > 0.05, * represents P < 0.05, ** represents P < 0.01, *** represents P < 0.001).

- 1. **Glucose Deprivation Triggers Neuroblastoma Disulfidptosis Potentiated by Cystine**
      Based on Professor Gan’s foundational research demonstrating that under glucose deprivation conditions, excessive accumulation of cystine can induce disulfidptosis in lung cancer cells (A549 cells)[1], we systematically investigated whether this metabolic vulnerability extends to neuroblastoma. Intriguingly, our study reveals a metabolic duality of cystine. It functions to sustain cell viability and promote proliferation under normoglycemic conditions (though the proliferative effect is attenuated at concentrations exceeding 400μM), while conversely promoting disulfide-dependent programmed cell death (disulfidptosis) in the context of glucose deprivation. Using CCK-8 assays, we observed that glucose deprivation alone induced significant cell death in neuroblastoma models (Figure S3A-B). This cytotoxic effect was markedly potentiated in a dose-dependent manner by cystine supplementation, however, the addition of cystine promoted cell growth under normal conditions. Notably, this combinatorial strategy exhibited consistent efficacy across both MYCN-amplified and non-amplified neuroblastoma subtypes, indicating broad applicability regardless of genetic heterogeneity. Strikingly, HK2 cells exhibited unique metabolic dependencies: cystine deprivation—but not glucose deprivation—induced robust cell death, regardless of glucose availability (Figure S3C). This phenomenon could not be attributed to SLC7A11 expression differences. Instead, their resistance to glucose deprivation likely stems from a metabolic preference for fatty acid β-oxidation[5], underscoring the necessity for cell type-specific NADPH depletion strategies. For neuroblastoma, glucose deprivation emerged as a viable approach, particularly given the kidney’s dual role as a common affected site and a chemotherapy-sensitive organ, thereby ensuring therapeutic specificity and safety. To confirm disulfidptosis as the underlying cell death mechanism, we conducted multi-layered analyses. Glucose deprivation alone reduced intracellular NADPH levels. Cystine co-treatment exacerbated this depletion in a dose-dependent manner. No significant NADPH changes were observed under standard glucose conditions (Figure S3D-E). SLC7A11 is the primary protein mediating cystine transportation[6]. In vitro characterization showed that neuroblastoma cell lines demonstrated significantly lower SLC7A11 protein abundance compared to A549 cells, yet maintained moderately higher expression than HEK293T embryonic kidney cells. SLC7A11 expression is independent of MYCN amplification, supporting the findings of [Hamed Alborzinia](https://pubmed.ncbi.nlm.nih.gov/?term=Alborzinia+H&cauthor_id=35484422) et al[7] (Figure S3F). Non-reducing Western blotting and cytoskeletal staining served as direct biochemical evidence of disulfidptosis[1, 8]. Parallel phalloidin staining revealed dose-dependent disintegration of actin networks – from partial cortical collapse under glucose restriction to complete cytoskeletal fragmentation with cystine supplementation (Figure S3G). Under disulfide stress conditions, covalent crosslinking in cytoskeletal proteins (e.g., FLNA, MYH9, Drebrin) prevents disulfide bond reduction in non-reducing electrophoresis, resulting in reduced electrophoretic mobility due to increased molecular weight. Consistent with this mechanism, glucose deprivation (Without the addition of exogenous cystine, the culture medium itself contains approximately 250 μM of cystine) ) induced discernible band shifts in these cytoskeletal regulators, indicative of basal disulfide crosslinking. Strikingly, cystine co-treatment (800 μM) dramatically exacerbated this phenomenon (Figure S3H-I). This mechanistic groundwork directly supports the development of glucose oxidase (GOx)-based nanotherapeutic strategies, wherein localized glucose depletion and cystine delivery can be spatially and temporally controlled to optimize disulfidptosis induction.


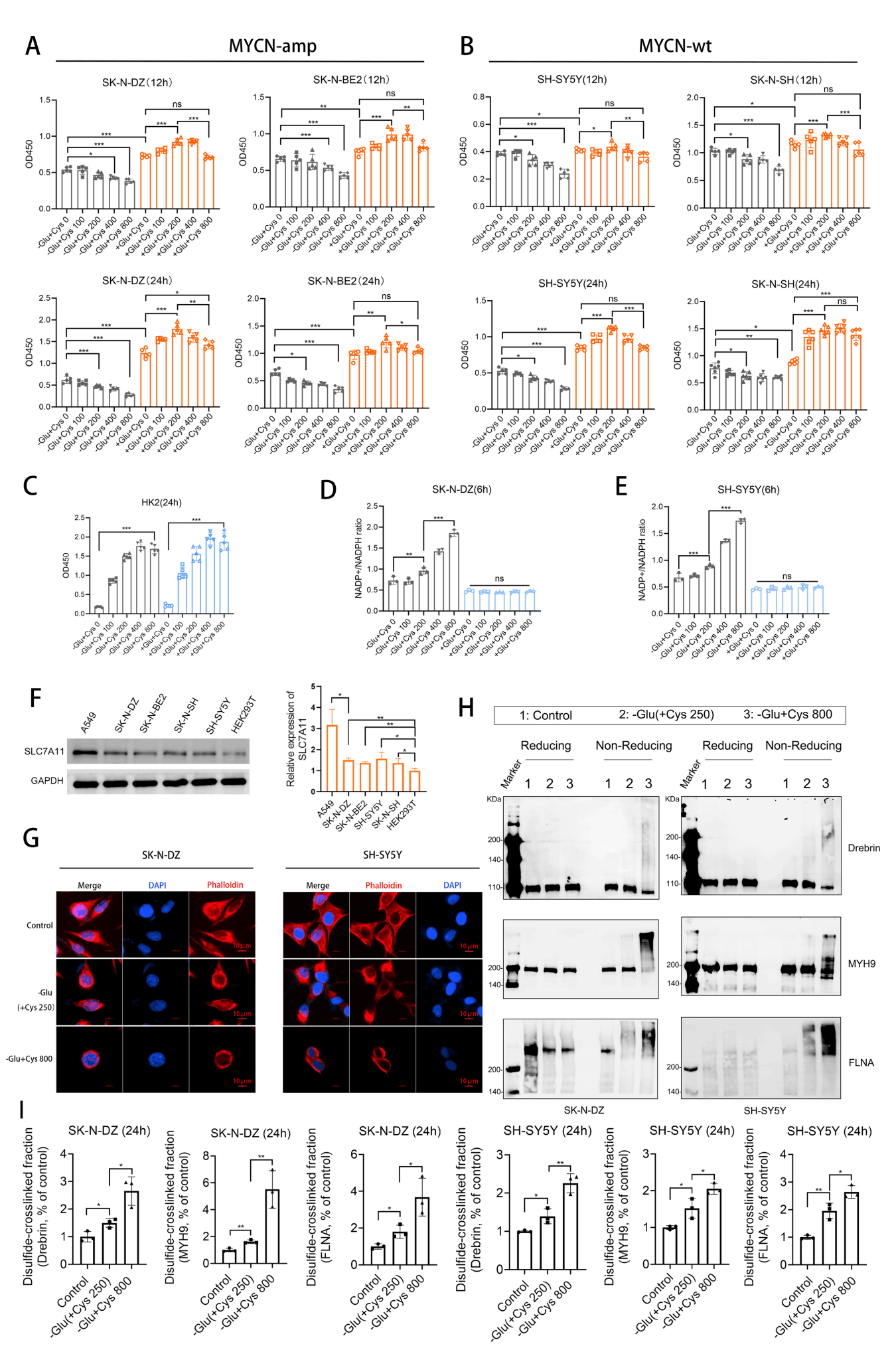


**Figure S3. Dual induction of disulfidptosis in neuroblastoma through glucose deprivation and cystine supplementation in vitro.** (A-C) CCK-8 viability assays demonstrating cystine-mediated cytotoxicity under glucose deprivation in: (A) MYCN-amplified neuroblastoma cell lines, (B) non-MYCN-amplified neuroblastoma cell lines, and (C) renal tubular epithelial cells (HK2) (n=5, mean ± SD). (D-E) NADPH depletion in SK-N-DZ (D) and SH-SY5Y (E) neuroblastoma cells under dual metabolic stress (n=3, mean ± SD) (F) Comparative SLC7A11 expression across cell lines: neuroblastoma models vs. high-expression control (A549) and low-expression baseline (HEK293T) (n=3, mean ± SD). (G) Phalloidin staining revealing cytoskeletal collapse characteristic of disulfidptosis.(H-I) Non-reducing western blot detecting aberrant disulfide-bonded protein aggregates (n=3, mean ± SD). Statistical comparisons by one-way ANOVA with appropriate post hoc tests; ns represents P > 0.05, * represents P < 0.05, ** represents P < 0.01, *** represents P < 0.001).

- 1. **Pro-proliferative effects of cystine under physiological conditions**

Although our work has revealed the metabolic duality of cystine—specifically, its role in promoting disulfidptosis under conditions of glucose deprivation—the mechanism by which it enhances cell proliferation under normoglycemic conditions remains unclear. Therefore, we proceeded to definitively characterize the function of cystine under normal glucose conditions. Flow cytometry showed a dose-dependent increase in the G2/M fraction with rising cystine; however, it declined at higher concentrations (≥400 µM) (Figure S4A-B). Immunoblotting yielded concordant results, with upregulation of PCNA and Cyclin B1 (Figure S4C-D). Conversely, 24-h cystine deprivation elicited mild early apoptosis, as detected by Annexin V/PI, although overall apoptosis remained limited (Figure S4E-F). Together, these data suggest that cystine both maintains viability and promotes proliferation in physiological redox states, but that excess cystine blunts this pro-growth effect.


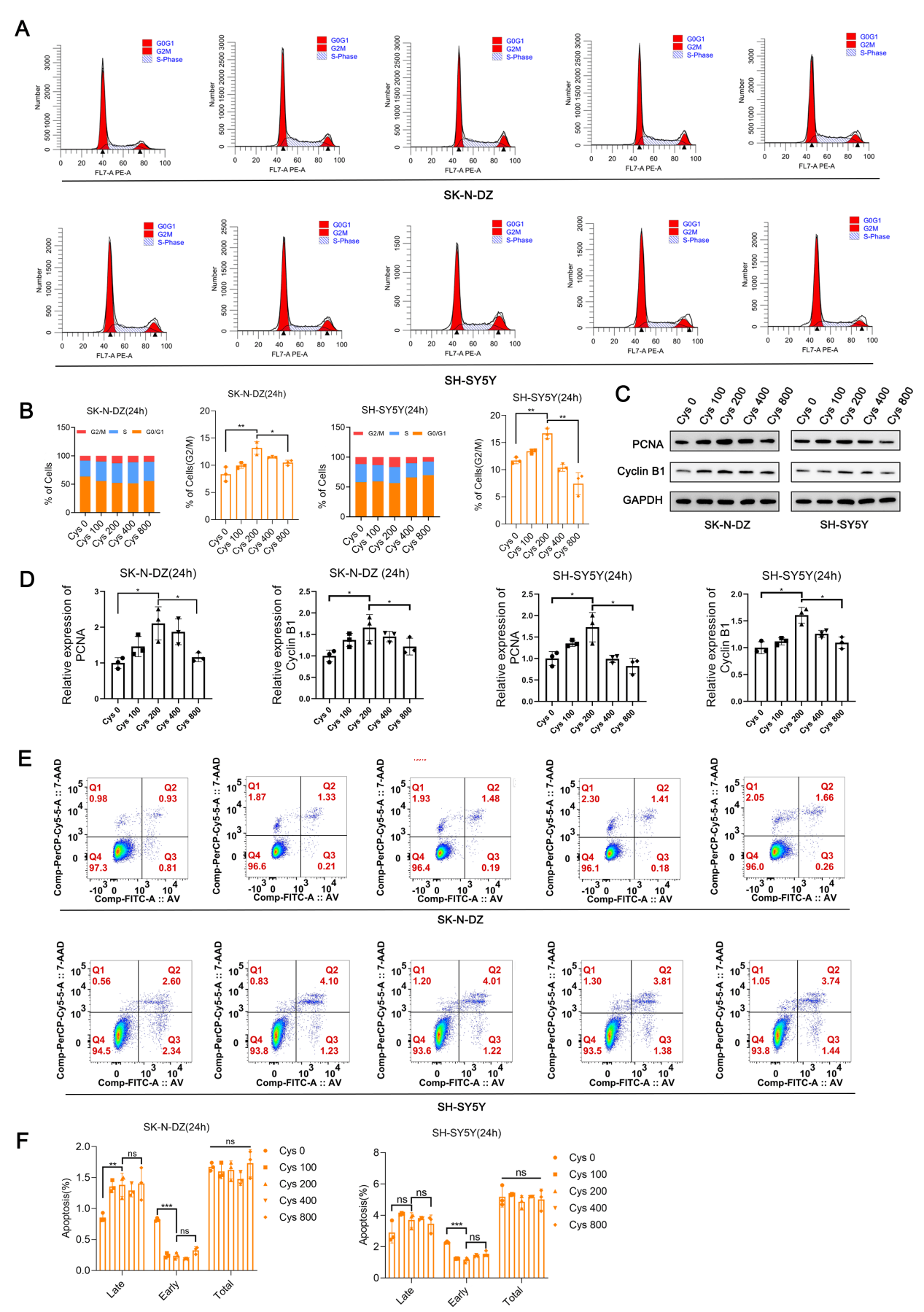


**Figure S4. Cystine promotes cell-cycle progression under normoglycemia with limited apoptosis.** Neuroblastoma cells (SK-N-DZ and SH-SY5Y) were cultured for 24 h in media containing the indicated cystine concentrations. (A) Representative PI DNA-content histograms showing cell-cycle phase distribution. (B) Comparison of Cell Cycle Phase Distribution and G2/M Phase Cell Percentages Across Groups (mean ± SD, n=3). (C) Immunoblots of PCNA and Cyclin B1 with GAPDH as a loading control. (D) Densitometric analysis of PCNA and Cyclin B1 (mean ± SD, n=3). (E) Representative Flow Cytometry Plots for Apoptosis Analysis after 24 h. (F) Pooled quantification of early, late, and total apoptosis (mean ± SD, n=3), showing mild early apoptosis upon cystine deprivation (Cys 0). Statistical comparisons by one-way ANOVA with Tukey post hoc tests; ns represents P > 0.05, ** represents P < 0.01, *** represents P < 0.001.

### **Synergistic Induction of Neuroblastoma Disulfidptosis by GOx and Cystine**

### To translate glucose deprivation-induced disulfidptosis into a viable in vivo therapeutic strategy, we explored the combinatorial application of GOx and cystine. Experiments were performed in both MYCN-amplified (SK-N-DZ) and non-amplified (SH-SY5Y) neuroblastoma cell lines. CCK-8 assays revealed the 24-hour IC₅₀ values of GOx to be 400.2 ng/ml and 322.2  ng/ml for SK-N-DZ and SH-SY5Y, respectively (Figure S5A). To evaluate the therapeutic efficacy of combined glucose oxidase (GOx) and cystine treatment, we subsequently applied GOx at its IC₅₀ concentration as the intervention dose. Cystine enhanced GOx-induced cytotoxicity in a dose-dependent manner (Figure S5B). Proliferation curves further demonstrated that GOx in combination with 800 μM cystine markedly suppressed cell proliferation,with significant inhibition observed as early as 8 hours post-treatment, and cell numbers falling below 10% of the control group by 48 hours (Figure S5C). Quantification of intracellular NADPH levels confirmed that the GOx–cystine co-treatment induced a substantial depletion of NADPH, supporting the proposed mechanism of disulfidptosis initiation (Figure S5D). Notably, we still observed a mild reduction in cell number under normal glucose conditions upon treatment with high concentrations of cystine, without affecting NADPH levels. We further validated direct evidence of disulfidptosis occurrence. Immunofluorescence imaging revealed cytoskeletal collapse—a morphological hallmark of disulfidptosis (Figure S5E) and protein crosslinking was observed (Figure S5F-G).


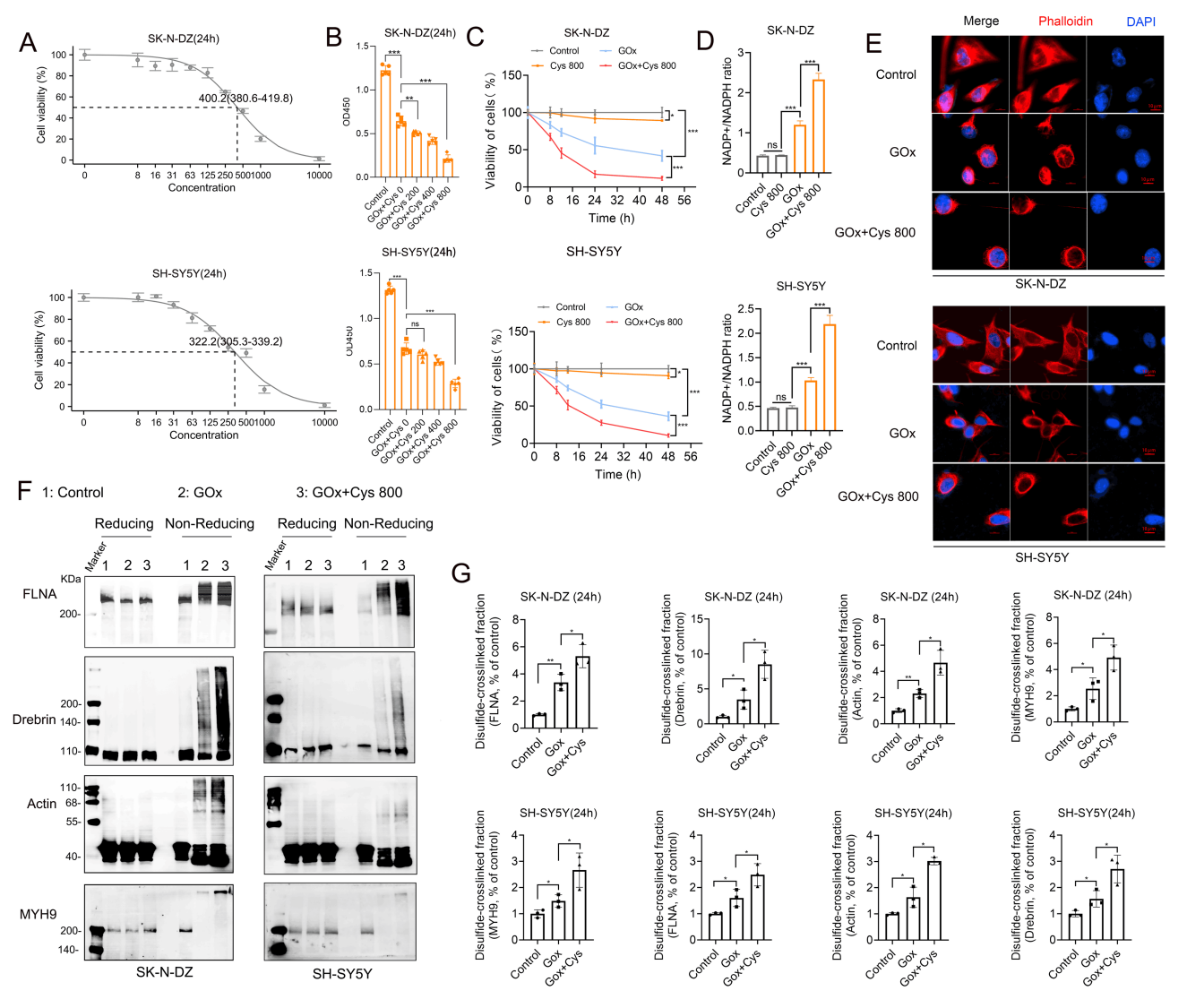


**Figure S5. Disulfidptosis induction through glucose oxidase (GOx) and cystine co-targeting in neuroblastoma.** (A) Dose-response profiling of GOx monotherapy in SK-N-DZ and SH-SY5Y cells using CCK-8 assays (IC50 determination) (mean ± SD, n=5). (B) Synergistic cytotoxicity of IC50-optimized GOx with cystine supplementation across neuroblastoma subtypes (mean ± SD, n=5). (C) Longitudinal proliferation kinetics under combinatorial metabolic perturbation (mean ± SD, n=5). (D) Stoichiometric shift in NADP+/NADPH ratio reflecting redox homeostasis breakdown (mean ± SD, n=3). (E) Phalloidin-based visualization of disulfidptosis-associated cytoskeletal disintegration. (F-G) Non-reducing Western blot detecting aberrant disulfide-bonded protein aggregates (mean ± SD, n=3). 4-parameter logistic fit for IC50 (A). one-way ANOVA with appropriate post hoc tests (B,D,G), Two-way ANOVA with Tukey’s post hoc (C); ns represents P > 0.05, * represents P < 0.05, ** represents P < 0.01, *** represents P < 0.001.

- 1. **Stability and Receptor-Mediated Internalization of the CD24-Targeted Nanoplatform**

To confirm sufficient GOx loading and preserved enzymatic activity, we quantified H_2_O_2_ production using an HRP-ABTS coupled assay. Time-dependent H_2_O_2_ profiles in the presence of 5.5 mM glucose showed low-level accumulation in the external medium, reflecting partial diffusion prior to MnO_2_ decomposition. Non-cystine formulations (MnO2@GOx@EM-CD24) exhibited minimal initial H_2_O_2_ (0.17±0.03 μM at 0.25 h), correlating with rapid O2 generation (12.80 mg/L at 0.5 h), followed by gradual increase to a plateau (2.21±0.01μM by 24–36 h) and slight decline (2.20±0.01μM at 48 h), consistent with O_2_ decay (7.13 mg/L at 6 h) due to catalyst fatigue. Cystine-modified variants maintained sustained low H_2_O_2_ (Cys-hMnO_2_@GOx@EM-CD24: 1.24±0.01 μM after 24 h; Cys-hMnO_2_@GOx@EM: 1.17±0.01μM after 24 h), aligning with stable O_2_ levels (≥9.5 mg/L over 6 h) through regulated catalysis via Mn-S coordination. These results affirm preserved GOx activity post-loading and sufficient loading for balanced metabolic intervention (Figure S6A). To assess colloidal stability under conditions mimicking systemic circulation, the nanocomposites were incubated in PBS containing 10% FBS at 37°C. Dynamic light scattering (DLS) measurements revealed that all formulations maintained hydrodynamic diameters with increases of less than 15% over 48 hours, alongside zeta potential variations of less than 10% (Figure S6B-C). This stability is attributed to the biomimetic exosome coating, which mitigates protein adsorption and aggregation. Cystine-modified variants exhibited marginally superior performance, likely due to enhanced structural integrity from the loaded cystine, consistent with the sustained catalytic activity observed in oxygen generation profiles (Figure 4K). Finally, we validated in vitro that CD24 modification enhances targeting and increases cellular uptake. Dual fluorescence labeling was employed to track the system: FITC-conjugated glucose oxidase (GOx) was encapsulated within the intermediate vesicular layer, while PKH26-labeled engineered membranes (EM) were anchored to the outer surface. After 6 hours of incubation, both Cys-hMnO_2_@GOx@EM-CD24 and non-targeted Cys-hMnO_2_@GOx@EM exhibited efficient cellular internalization, with co-localization observed between FITC-GOx and PKH26 signals (Figure S6F-G). The concurrent intracellular presence of both outer EM and intermediate-layer GOx indirectly confirmed the successful delivery of the innermost Cys-MnO₂ payload. Notably, Cys-hMnO_2_@GOx@EM-CD24 showed significantly higher cellular uptake compared to non-targeted vesicles, attributable to antibody–antigen interaction-mediated endocytosis (Figure S6D-G)


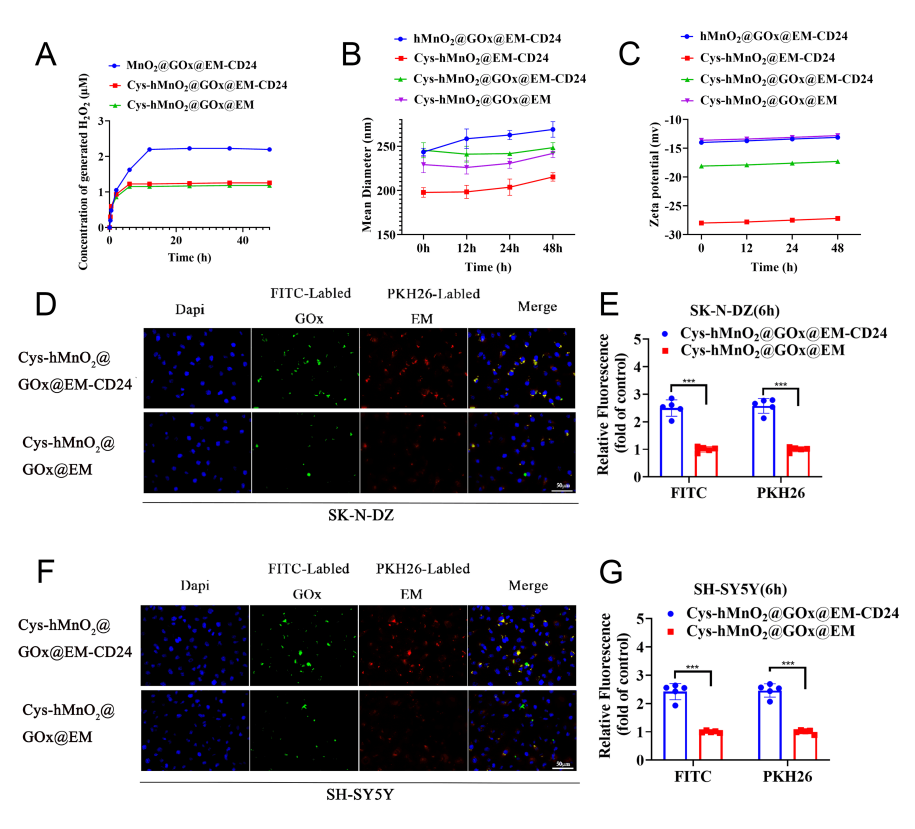


**Figure S6. H₂O₂ generation, colloidal stability, and CD24-mediated cellular uptake of the nanosystem.** (A) Time course of H₂O₂ released from GOx-loaded formulations (mean, n=3,). (B–C) Hydrodynamic diameter (B) and zeta potential (C) of the indicated nanoparticles after incubation in PBS (pH 7.4) containing 10% FBS at 37 °C for 0–48 h, showing stable size and surface charge (mean ± SD, n=3). (D, F) Representative fluorescence micrographs after 6 h incubation with SK-N-DZ (D) or SH-SY5Y (F). (E, G) Quantification of cellular fluorescence for FITC and PKH26 was obtained by microplate spectrofluorometry from parallel wells, reported as fold of the non-targeted control after background subtraction (mean ± SD; n = 5). Statistics: unpaired two-tailed t-test.

- 1. **Cys-hMnO_2_@GOx@EM-CD24 Induces Redox Homeostasis Disruption**

To delineate redox imbalance, we measured intracellular ROS and the GSH/GSSG ratio. ROS imaging (Figure S7A-B) and quantification (Figure S7C) at 6 h showed marked ROS elevation in Group 5. Consistently, GSH/GSSG analysis (Figure S7D) revealed profound GSH depletion and a significant fall in the GSH/GSSG ratio in Groups 4, 5 and 6, supporting GOx-generated H₂O₂ as the principal driver of oxidative stress. Notably, despite similarly high ROS in Group 6, its disulfidptosis readouts remained lower than those of Group 5, underscoring the importance of CD24-mediated uptake and intracellular delivery for maximizing the catalytic cascade and overall cytotoxicity. Meanwhile, the addition of cystine enhanced the production of ROS to a certain extent.

We assessed each formulation’s ability to trigger disulfidptosis by quantifying the disulfide-linked fraction of key cytoskeletal proteins—FLNA, MYH9, and Drebrin—using non-reducing immunoblotting (FigureS7 E). The complete nanocascade (Group 5) produced the highest and statistically significant increases in FLNA, MYH9, and drebrin crosslinking compared with the vehicle control and the GOx-omitted formulations. These data indicate that GOx-driven glucose oxidation is indispensable for initiating disulfidptosis, and that cystine loading together with CD24 targeting act cooperatively to amplify the response.


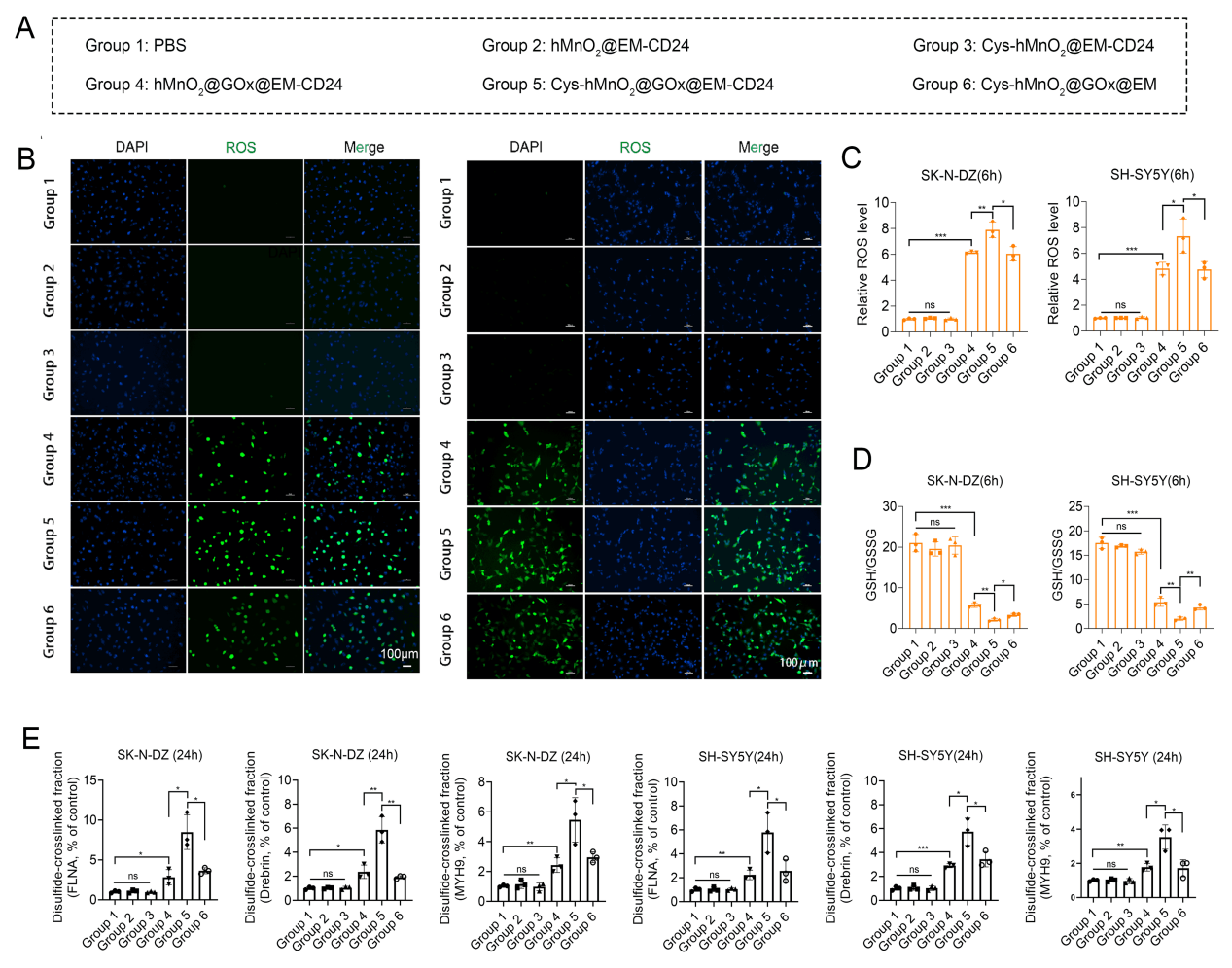


Figure S7. Induces Disulfidptosis by Disrupting Intracellular Redox Homeostasis (A) Schematic illustration of the six treatment groups. (B) Microscopy images showing ROS generation after treatment. (C) Quantitative analysis of relative intracellular ROS levels in cells after treatment (mean ± SD, n=3). (D) Intracellular GSH/GSSG ratio (mean ± SD, n=3). (E) Quantification of disulfide-crosslinked fractions of cytoskeletal proteins (mean ± SD, n=3). Statistical comparisons by one-way ANOVA with Tukey’s post hoc tests; ns represents P > 0.05, * represents P < 0.05, ** represents P < 0.01, *** represents P < 0.001.

- 1. **Comparative assessment of the therapeutic efficacy and adverse effects of Cys-hMnO₂@GOx@EM-CD24**

To further highlight the therapeutic advantage of Cys-hMnO_2_@GOx@EM-CD24, we compared it with the clinically used chemotherapeutic doxorubicin (Dox); during treatment, no significant body-weight changes were observed across groups (Figure S8A). Cys-hMnO₂@GOx@EM-CD24 achieved the greatest tumor control, yielding significantly lower tumor weights than both PBS and Dox (Figure S8B), consistent with the reduced tumor burden on H&E (Figure 8C). Systemically, Dox induced mild biochemical evidence of injury (increased CK, ALT/AST, and Urea), whereas dEM-CD24 remained indistinguishable from PBS (Figure S8D). Likewise, hepatic and renal redox homeostasis was preserved with dEM-CD24 (GSH/GSSG and MDA near PBS levels), while Dox elevated oxidative-stress readouts (Figure S8E). Together, these data indicate that Cys-hMnO_2_@GOx@EM-CD24 provides superior antitumor efficacy with a more favorable safety profile than doxorubicin.

**
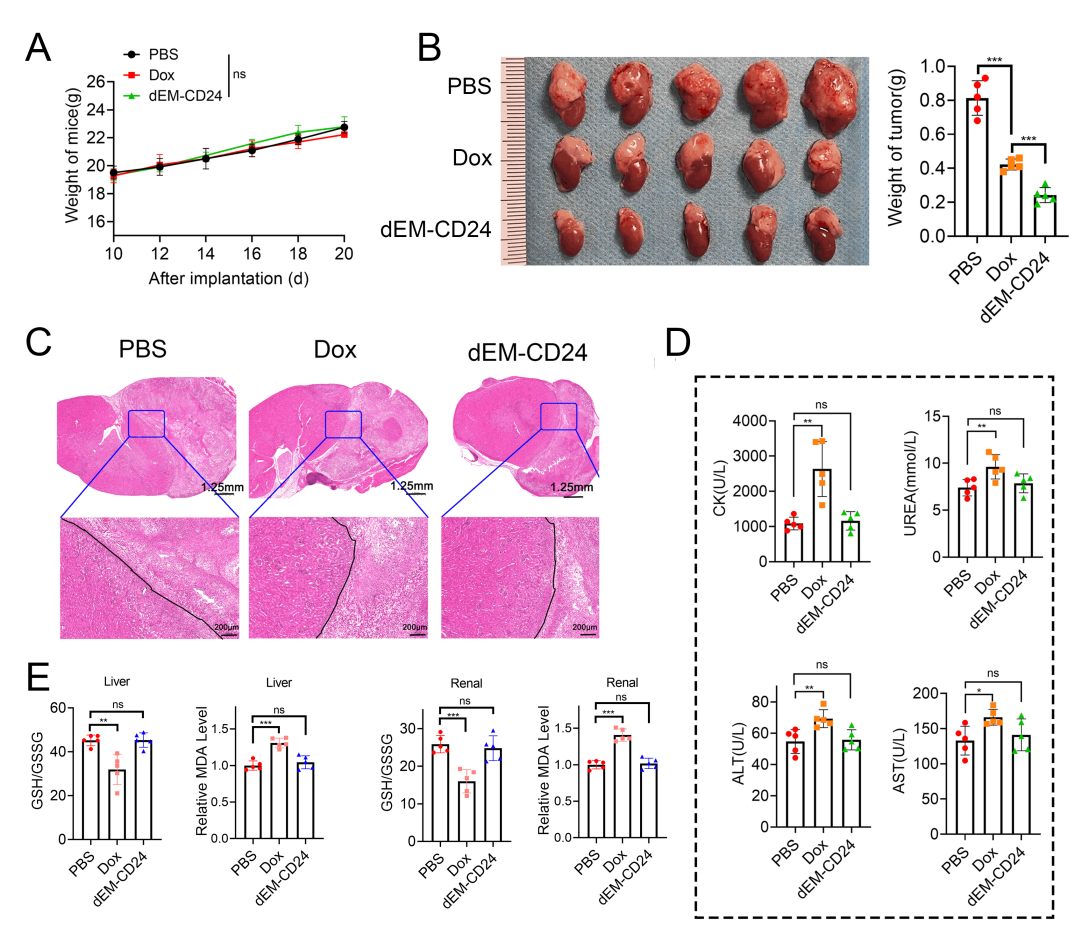
**

**Figure S8. Comparative assessment of the therapeutic efficacy and adverse effects of Cys-hMnO₂@GOx@EM-CD24.** (A) Body-weight trajectories during treatment (n = 5 mice/group, mean ± SD). (B) Representative photographs of excised tumors and endpoint tumor weight (n = 5 mice/group, mean ± SD). (C) H&E staining of orthotopic tumors. (D) Serum biochemistry—CK, Urea, ALT, AST—after treatment (n = 5 mice/group, mean ± SD. (E) Hepatic and renal oxidative-stress indices—GSH/GSSG ratio and MDA level (n = 5 mice/group, mean ± SD). Body weight: repeated-measures ANOVA. Other group comparisons: one-way ANOVA with Tukey post hoc; ns represents P > 0.05, * represents P < 0.05, ** represents P < 0.01, *** represents P < 0.001.

**2.9. Cys-hMnO2@GOx@EM-CD24 Suppress expression of PCNA and MMP2**

We assessed the expression of proliferation marker PCNA and invasion-associated protein MMP2 in tumor tissues from the orthotopic tumor model, both of which were significantly suppressed following treatment with Cys-hMnO_2_@GOx@EM-CD24 (Figure S9).


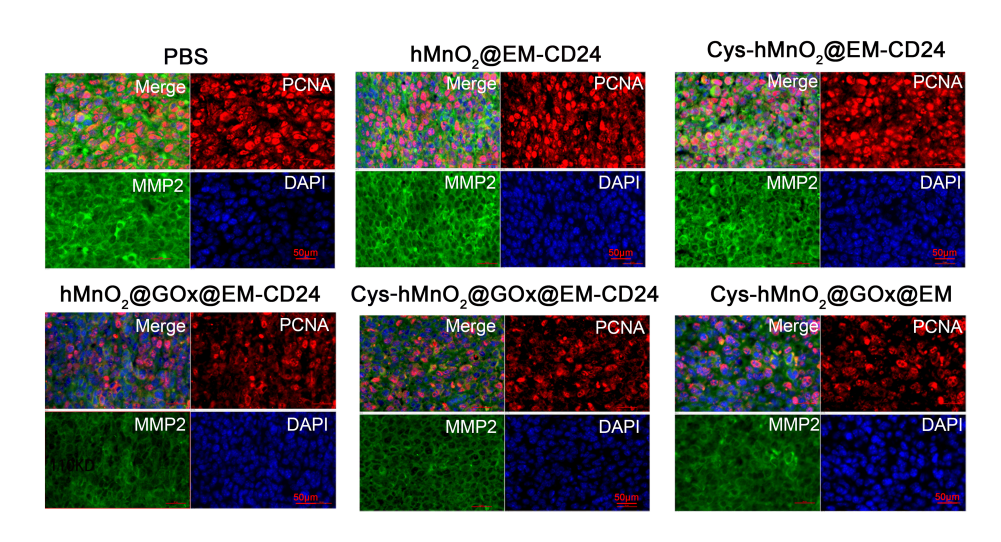


**Figure S9: Cys-hMnO_2_@GOx@EM-CD24 Suppress expression of PCNA and MMP2.** Immunofluorescence co-staining showed reduced expression of proliferation marker PCNA and invasion-associated marker MMP2 in the treatment group.

**2.10. Safety Profile of Cys-hMnO_2_@GOx@EM-CD24 in nervous system**

Because CD24 is enriched in the developing nervous system, we first examined potential neurotoxicity in vivo. H&E staining of brains from orthotopic tumor–bearing mice treated with Cys-hMnO₂@GOx@EM-CD24 versus PBS revealed no overt neuropathology (Figure S10A). We then evaluated NGF-differentiated PC12 neuron-like cells (Figure S10B). Western blotting showed that, although CD24 was detectable in PC12-derived neuron-like cells, its expression was lower than in neuroblastoma cell lines (SK-N-DZ, SH-SY5Y), and SLC7A11 was likewise low (Figure S10C–E). Together with the richer tumor vasculature in vivo, this difference supports a CD24-targeting advantage in tumors. Functionally, when NGF-differentiated PC12 cells were exposed to Cys-hMnO₂@GOx@EM-CD24 at doses approximating the neuroblastoma IC50, they retained 80-90% viability. The IC50 in PC12-derived neuron-like cells was ~36 μg/mL, yielding selectivity indices of 3.7 and 4.7 relative to SK-N-DZ and SH-SY5Y, respectively.


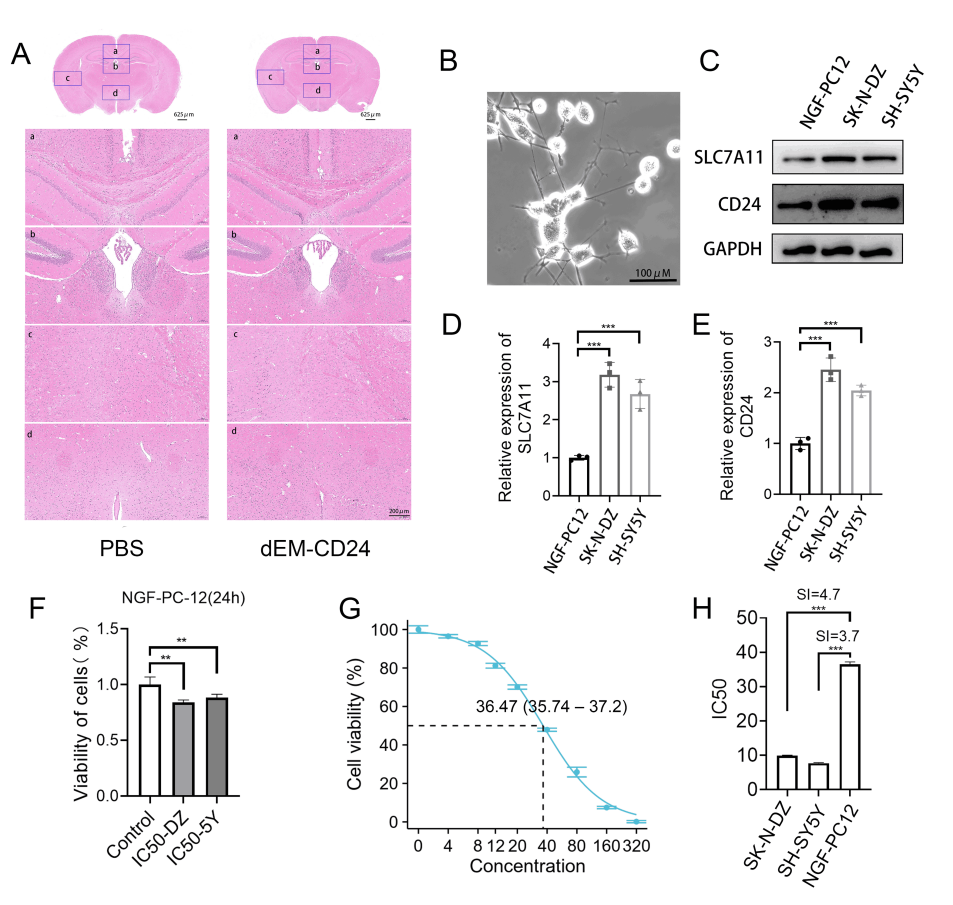


**Figure S10:** **Neurotoxicity and systemic safety of the CD24-targeted nanoplatform.**
(A) H&E-stained coronal brain sections from PBS vs. Cys-hMnO₂@GOx@EM-CD24 (dEM-CD24) showing no overt neuropathology. (B) NGF-differentiated PC12 neuron-like cells (NGF-PC12) in culture (72h). (C) Western blots of SLC7A11 and CD24 in NGF-PC12, SK-N-DZ, and SH-SY5Y. (D–E) Densitometric quantification of SLC7A11 and CD24 ((mean ± SD, n=3). (F) NGF-PC12 viability after 24 h exposure to the nanoplatform at tumor-cell IC50 doses (SK-N-DZ IC50; SH-SY5Y IC50) (mean ± SD, n=3). (G) Dose–response curve of tumor-cell viability with IC50 indicated (mean ± SD, n=5 ). (H) IC50 values and selectivity indices(SI) for PSN vs tumor cells. Statistical comparisons by one-way ANOVA with Tukey’s post hoc (D,E,F, H); log-logistic for G; ns represents P > 0.05, * represents P < 0.05, ** represents P < 0.01, *** represents P < 0.001).

**References**

1. Liu, X., et al., *Actin cytoskeleton vulnerability to disulfide stress mediates disulfidptosis.* Nat Cell Biol, 2023. **25**(3): p. 404-414.

2. Mi, T., et al., *Doxycycline hydrochloride inhibits the progress of malignant rhabdoid tumor of kidney by targeting MMP17 and MMP1 through PI3K-Akt signaling pathway.* Eur J Pharmacol, 2024. **964**: p. 176291.

3. Sun, F., et al., *Anti-CD24 Antibody-Nitric Oxide Conjugate Selectively and Potently Suppresses Hepatic Carcinoma.* Cancer Res, 2019. **79**(13): p. 3395-3405.

4. Guo, P., et al., *Therapeutic Targeting of Osteosarcoma and Lung Metastases in Preclinical Models Using a CD24 Antibody-Drug Conjugate.* Advanced Therapeutics. **n/a**(n/a): p. 2400423.

5. Liu, L., et al., *Twist1 downregulation of PGC-1α decreases fatty acid oxidation in tubular epithelial cells, leading to kidney fibrosis.* Theranostics, 2022. **12**(8): p. 3758-3775.

6. Yan, Y., et al., *SLC7A11 expression level dictates differential responses to oxidative stress in cancer cells.* Nat Commun, 2023. **14**(1): p. 3673.

7. Alborzinia, H., et al., *MYCN mediates cysteine addiction and sensitizes neuroblastoma to ferroptosis.* Nat Cancer, 2022. **3**(4): p. 471-485.

8. Wu, S., et al., *Glucose deprivation-induced disulfidptosis in human nucleus pulposus cells: a novel pathological mechanism of intervertebral disc degeneration.* Biol Direct, 2024. **19**(1): p. 81.
